# Sustained mucosal colonization and fecal metabolic dysfunction by *Bacteroides* associates with fecal microbial transplant failure in ulcerative colitis patients

**DOI:** 10.1038/s41598-024-62463-8

**Published:** 2024-08-09

**Authors:** Bing Zhang, Kevin M. Magnaye, Emily Stryker, Jacqueline Moltzau-Anderson, Cara E. Porsche, Sandra Hertz, Kathryn E. McCauley, Byron J. Smith, Martin Zydek, Katherine S. Pollard, Averil Ma, Najwa El-Nachef, Susan V. Lynch

**Affiliations:** 1 Department of Medicine, Division of Gastrointestinal and Liver Diseases, Keck School of Medicine, University of Southern California, Los Angeles, CA 90033 USA; 2 Division of Gastroenterology, Department of Medicine, University of California San Francisco, San Francisco, CA 94143 USA; 3 The Benioff Center for Microbiome Medicine, University of California San Francisco, San Francisco, CA 94143 USA; 4 Department of Infectious Diseases, Aalborg University Hospital, Aalborg, Denmark; 5 The Gladstone Institutes, Data Science and Biotechnology, San Francisco, CA 94158 USA; 6 Department of Epidemiology and Biostatistics, University of California San Francisco, San Francisco, CA 94158 USA; 7 Chan Zuckerberg Biohub, San Francisco, CA University of California, San Francisco, CA 94158 USA; 8 Division of Gastroenterology, Henry Ford Health System, Detroit, MI 48208 USA

**Keywords:** Ulcerative colitis, Microbiome

## Abstract

Fecal microbial transplantation (FMT) offers promise for treating ulcerative colitis (UC), though the mechanisms underlying treatment failure are unknown. This study harnessed longitudinally collected colonic biopsies (n = 38) and fecal samples (n = 179) from 19 adults with mild-to-moderate UC undergoing serial FMT in which antimicrobial pre-treatment and delivery mode (capsules versus enema) were assessed for clinical response (≥ 3 points decrease from the pre-treatment Mayo score). Colonic biopsies underwent dual RNA-Seq; fecal samples underwent parallel 16S rRNA and shotgun metagenomic sequencing as well as untargeted metabolomic analyses. Pre-FMT, the colonic mucosa of non-responsive (NR) patients harbored an increased burden of bacteria, including *Bacteroides*, that expressed more antimicrobial resistance genes compared to responsive (R) patients. NR patients also exhibited muted mucosal expression of innate immune antimicrobial response genes. Post-FMT, NR and R fecal microbiomes and metabolomes exhibited significant divergence. NR metabolomes had elevated concentrations of immunostimulatory compounds including sphingomyelins, lysophospholipids and taurine. NR fecal microbiomes were enriched for *Bacteroides fragilis* and *Bacteroides salyersiae* strains that encoded genes capable of taurine production. These findings suggest that both effective mucosal microbial clearance and reintroduction of bacteria that reshape luminal metabolism associate with FMT success and that persistent mucosal and fecal colonization by antimicrobial-resistant *Bacteroides* species may contribute to FMT failure.

## Introduction

Inflammatory bowel disease (IBD) is a chronic, relapsing gastrointestinal inflammatory disease primarily comprised of Crohn’s disease (CD) and ulcerative colitis (UC). In 2015, the Center for Disease Control reported that about 1.3% of U.S. adults (3.1 million people) had received a diagnosis of IBD^1^. While IBD was relatively rare in developing countries in the twentieth century, over the past two decades it has become widespread globally with rising prevalence particularly in newly industrialized nations^2^. Thus, there is an urgent need to develop effective therapies for this patient population.

While surgical colectomy remains the only cure for UC^3^, standard treatment for disease management include antibiotics, corticosteroids, immunomodulators, biological agents, and small molecules that exhibit varying efficacy^4^ and can lead to risk of immunosuppression, loss of response over time, and development of anti-drug antibodies^5,6^. The exact etiology of UC is unclear; however, an altered gut microbiome composition and function is known to play a role in the development and pathogenesis of the disease^7–12^. Thus, therapeutics specifically targeting the gut microbiome have gained interest, with fecal microbial transplants (FMT), particularly repeatedly administered FMTs, demonstrating promise for the treatment of UC^13,14^. The number of UC FMT trials performed to date has permitted meta-analyses which indicate that approximately half of UC patients who receive FMT respond to treatment (defined by most studies as Mayo score decrease ≥ 3), with one-third achieving clinical remission of their symptoms (defined by most studies as Mayo score ≤ 2)^15,16^. These clinical response rates surpass those reported in phase-III clinical trials for the biological agents golimumab and vedolizumab that are commonly used to treat the condition^17^. However, the mechanisms underlying FMT responsiveness and, of parallel importance, lack of responsiveness in UC patients remain unknown and represents a significant barrier to the development of more effective, tailored microbial-based therapies for this patient population.

Large cohort studies have demonstrated modifiable environmental risk factors contributing to approximately 50% of IBD disease prevalence, many of which may impact disease pathogenesis via modulation of the gut microbiome^18,19^. Although several murine models of colitis have been employed to study IBD, none entirely capture the complexities of human UC and CD or environmental exposures associated with these conditions^20,21^. Despite these limitations, studies utilizing murine models of colitis have confirmed a role for the gut microbiome in both the promotion and resolution of UC disease symptoms. However, little is known of the specific microbial strains and products responsible for immune modulation and clinical disease remission in UC patient populations. Recently, studies of human populations have shown that microbial-derived primary and secondary metabolites are a means by which intestinal microbiomes modulate immune function^10,22,23^, though their role in the remission of UC symptoms remains largely unknown.

Here, we leveraged paired mucosal biopsy and fecal specimens collected longitudinally during a human FMT interventional trial of UC patients, to identify mechanisms by which microbial species and their metabolic products may promote clinical remission or treatment non-responsiveness. We show that effective anti-microbial pre-treatment of the intestinal mucosa improves treatment responsiveness, and that fecal microbial composition, function and metabolic productivity post-treatment stratifies by clinical outcome. Though based on a small human clinical trial, these longitudinally collected data provide the first indications that persistent colonization by antimicrobial resistant mucosal microbes may underlie FMT failure. The data also indicate that re-introduction of luminal bacterial strains with the capacity to both consume inflammatory metabolites and produce anti-inflammatory molecules associates with clinical responsiveness to FMT, suggesting that both mucosal and luminal microbiomes play key roles in treatment efficacy.

## Results

### Demographic and clinical characteristics of trial participants

**Figure 1:**
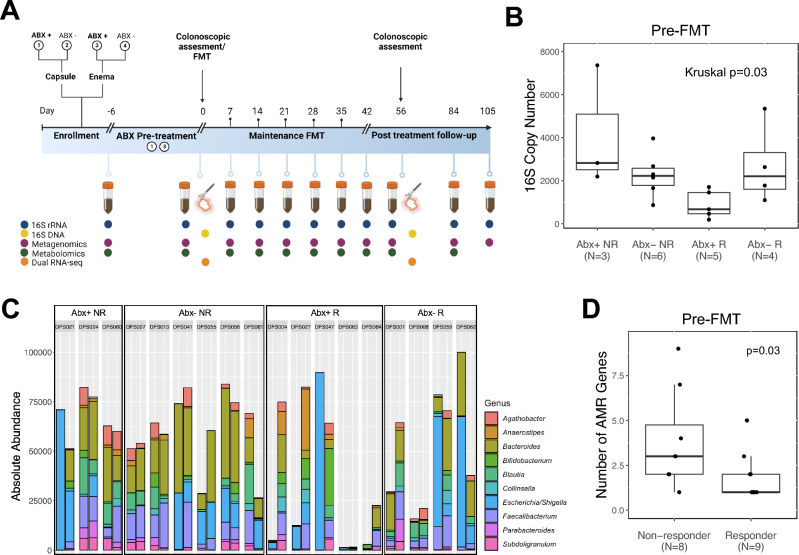
Antibiotic-pretreated FMT-responsive patients are depleted of transcriptionally active bacteria on the colonic mucosa. (**A**) Schematic of the study design, clinical arms, and biospecimen collections. Participants were randomized to one of four arms: (1) antibiotic-pretreated with capsule delivery; (2) non-antibiotic-pretreated with capsule delivery; (3) antibiotic-pretreated with enema delivery; (4) non-antibiotic-pretreated with enema delivery. Initial FMT was delivered by colonoscopy, followed by weekly FMTs over the subsequent 6 weeks. (**B**) Bacterial burden based on 16S rRNA copy number measured by qPCR for four antibiotic response groups: antibiotic-pretreated non-responders (Abx+ NR), non-antibiotic-pretreated non-responders (Abx− NR), antibiotic-pretreated responders (Abx+ R), and non-antibiotic-pretreated responders (Abx− R). (**C**) Absolute abundance plots of the 16S rRNA read counts for the top 10 genera are displayed for the four antibiotic/response groups. Columns indicate individual patients (with unique study ID) with the pre-FMT (after antibiotic pretreatment) sample displayed on the left and the post-FMT sample on the right. (**D**) The number of microbial-encoded antimicrobial resistance (AMR) genes expressed by mucosal-associated microbiota in NR and R patients (One-sided Wilcoxon rank sum test).

Thirty patients were enrolled and randomized into four clinical arms (with or without antibiotic pretreatment and FMT delivery either via capsule or enema; Fig. 1A), of whom nineteen completed the study and were included in our analysis (Table S1). Eleven participants withdrew from the study, four due to disease worsening, one due to poor tolerance of oral capsules, four due to the SARS-CoV-2 shutdown and two for personal reasons. The nineteen participants who completed the study were treated with seven successive FMTs and were assessed post-treatment; ten participants met the study’s primary outcome of clinical response to FMT, defined as a decrease in ≥ 3 points from their pre-treatment Mayo score by the conclusion of the study (Week 56, or F/u 2; Fig. 1A). This time point is intended to parallel that of large Phase 3 clinical drug trials, which report clinical improvement over 52 weeks^24^. There were no significant differences in baseline demographics, extent of disease, erythrocyte sedimentation rate, C-reactive protein, or concomitant medication use between responders (R) and non-responders (NR; p < 0.05; Table 1). However, at baseline prior to treatment, the NR group had significantly higher fecal calprotectin (Wilcoxon p = 0.007) compared with the R group, indicating elevated intestinal inflammation at baseline in those who failed to respond to FMT. No significant differences in clinical response rates were observed based on antibiotic exposure, mode of FMT delivery, or study arm although it was notable that all four patients who received antibiotics and FMT via capsules responded to treatment.

**Table 1:** Demographic, disease, and clinical trial characteristics of patients at time of enrollment by response group.

	Non-responder (N = 9)	Responder (N = 10)	P-value
Demographics			
Gender (% female)	44.4	30	0.86
Age (mean year ± SD)	38.3 ± 10.4	43.0 ± 13.5	0.41
Clinical measures			
Biologic therapy (%)	33.3	40.0	1.00
Steroid therapy (%)	22.2	20.0	1.00
Years since diagnosis (mean ± SD)	8.78 ± 6.14	13.3 ± 12.1	0.32
Extent of disease (left/right/transverse)	3/6/0	4/4/2	0.29
Total mayo score (mean ± SD)	6.22 ± 1.39	5.90 ± 1.37	0.62
Median ESR (min, max)	18 (5, 35)	7 (1, 45)	0.44
Median CRP (min, max)	2.50 (1, 14)	2.75 (0.2, 54)	0.39
Median fecal calprotectin (min, max)	993 (16, 2000)	182 (16, 751)	0.007
Clinical arms			
Antibiotic administration (Y/N)	6/3	4/6	0.37
FMT delivery (C/E)	3/6	6/4	0.37
Arm (Abx+,C/Abx−,C/Abx+,E/Abx−,E)	0/3/3/3	4/2/2/2	0.24

Clinical response defined as a decrease ≥ 3 in Mayo score by the end of the clinical trial.

ESR, Erythrocyte sedimentation rate; CRP, C-reactive protein; C, capsule; E, enema; Abx, antibiotics. Continuous variables were tested with a Wilcoxon rank sum test and categorical variables were tested using a Fisher’s exact test.

### Colonic microbial clearance with antimicrobials associates with clinical response to FMT

Previous reports indicate that antimicrobial treatment pre-FMT may enhance clinical response in patients with UC^25–27^. Use of antibiotics prior to FMT can decrease both the microbial burden and competition for the newly introduced species, and this strategy has been employed in FMT studies beyond IBD^28,29^. A combination of vancomycin, metronidazole and neomycin was administered for three days to patients randomized to antimicrobial pre-treatment groups. These antimicrobials were selected based on their combined comprehensive coverage of Gram-positive (specifically *Clostridioides difficile*), anaerobic, and Gram-negative bacteria. We thus examined whether antimicrobial pretreatment influenced mucosal-associated bacteria in R and NR patient groups. Since DNA-based quantification methods do not differentiate between live and dead microbes, we sought to overcome this limitation by analyzing transcriptionally active mucosal-associated bacteria. Specifically, RNA was extracted from mucosal biopsies, confirmed DNA-free and used to produce cDNA to quantify 16S rRNA copy number from mucosal biopsies collected before (N = 18) or after (N = 19) FMT. Of note, the pre-FMT biopsy was collected following antibiotic administration from those patients in the antibiotic pre-treatment groups (Abx+). FMT-responsive patients pre-treated with antibiotics (Abx+ R) exhibited decreased mucosal 16S rRNA copy number pre-FMT compared with either responders who did not receive antibiotics (Abx− R) or with non-responders who did (Abx+ NR or Abx− NR groups; Kruskal p = 0.03; Figs. 1B; S1A). Mucosal bacterial copy number did not differ between the four groups at the post-FMT time point (Kruskal p = 0.67; Fig. S1B), suggesting that the types, rather than the burden of bacteria colonizing the colonic mucosa post-FMT relate to clinical response.

To assess differences in composition of the transcriptionally active mucosal-associated microbiota, we generated 16S rRNA V4 amplicon sequence profiles from the same cDNA pool used to quantify mucosal bacterial burden. The number of 16S rRNA sequences per sample significantly correlated with the quantity of mucosal bacteria present (as determined by 16S rRNA copy number; N = 37; Spearman’s p = 1.65 × 10^–6^; ρ = 0.72; Figs. 1C, S1C and D) indicating that the burden of transcriptionally active mucosal-associated bacteria is reflected by 16S rRNA amplicon sequencing. A comparison of change in mucosal-associated 16S rRNA burden (copy number) from pre- to post-FMT indicated that the NR group exhibited a lower degree of change compared with the R group (Wilcoxon p = 0.01; Fig. S2A). The R group, especially those who received antibiotics (Abx+ R; Fig. S2B), exhibited a significant (Kruskal p = 0.03) increase in mucosal bacterial burden post-FMT, indicative of successful re-population of the mucosal microbiota with viable bacteria following both antibiotic pretreatment and FMT.

Based on these observations, we next determined whether the mucosal-associated microbiota of NR patients exhibit increased expression of antimicrobial-resistance (AMR) determinants using IDSeq^30^. Irrespective of antimicrobial pre-treatment, NR patients expressed a significantly greater diversity of mucosal-associated microbial AMR genes compared to the R group pre-FMT (Figs. 1D; S3; Wilcoxon p = 0.03). We next examined these AMRs for transcripts that confer resistance to the antibiotics used in the pre-treatment cocktail (vancomycin, neomycin, and metronidazole). Five (of 30) AMR genes (*Aph3-la, Aac3-l, Aac3-lb, AacAad*, and *Aac3-lla*) were detected in NR patients, all of which are known to confer resistance to neomycin (Table S2).

**Figure 2:**
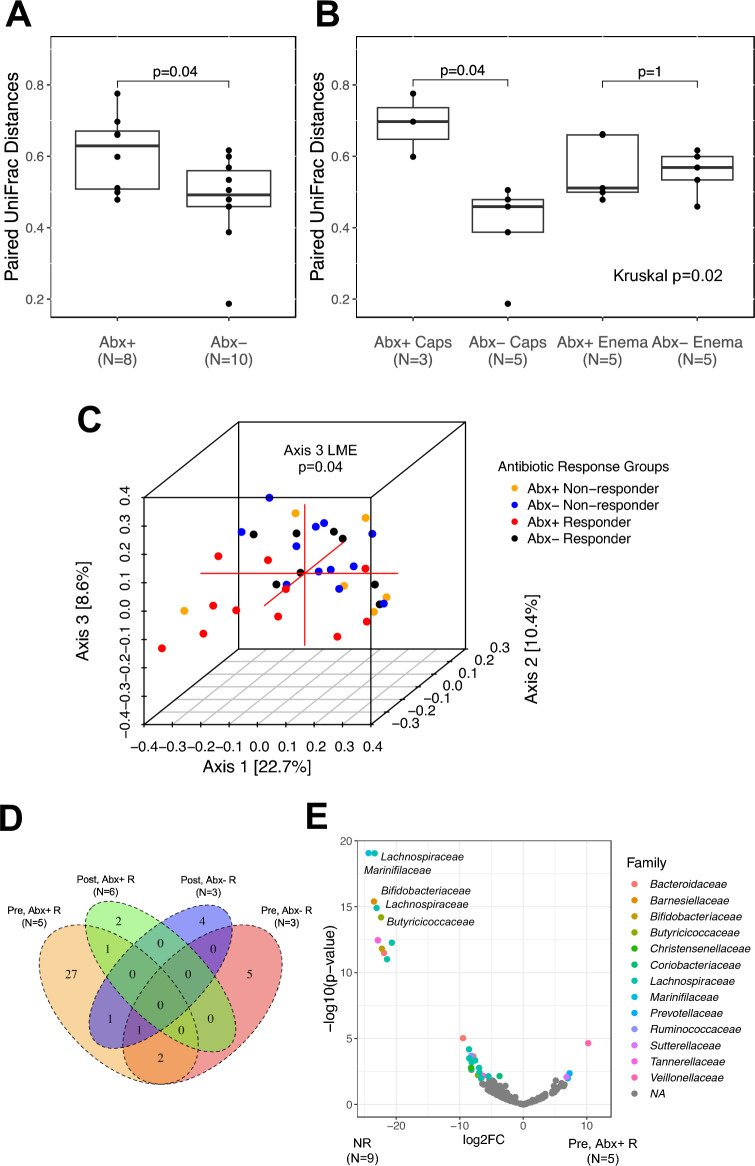
Antibiotic-pretreated FMT-responsive patients have a distinct profile of mucosal-associated transcriptionally active bacteria. Paired Unweighted UniFrac distances between pre-FMT and post-FMT samples are stratified by (**A**) antibiotic pretreatment and (**B**) clinical arm. (**C**) Principal Coordinates Analysis plot of mucosal -associated transcriptionally active bacteria shows all samples (pre-FMT and post-FMT) colored by antibiotic response group. Associations between antibiotic response group and the first three axes were tested with a linear mixed effects model, adjusting for patient. Axis 3 was significantly associated with antibiotic response groups. (**D**) Four different subsets of FMT-responsive patients (stratified by time point and antibiotic pretreatment) were compared to all non-responders. Numbers of differentially abundant taxa are displayed in the Venn diagram (DESeq2; FDR-adjusted p < 0.05). (**E**) Compared to non-responders, twenty-nine and three bacteria were decreased and increased, respectively, in antibiotic-pretreated responders (Pre, Abx+ R) prior to FMT. See Fig. S5 for volcano plots for all four tests of differential abundance. Abx, antibiotic; Caps, capsule; FMT, fecal microbial transplantation; R, responders; NR, non-responders.

We next related pairwise mucosal-associated transcriptionally active bacterial profiles pre- and post-FMT, to study variables. There was no difference in pairwise distances based on treatment response (Wilcoxon, P = 0.56) or mode of FMT delivery (capsule vs enema; Wilcoxon, P = 0.46), indicating that these factors alone do not significantly relate to the degree of change in the mucosal-associated microbiota over time. However, a significant difference was observed based on antibiotic pretreatment (Wilcoxon p = 0.04; Fig. 2A), with those administered antibiotics exhibiting a significantly greater distance (dissimilarity) in paired mucosal microbiota pre- and post-FMT. Pairwise distances also significantly differed between study arms (Kruskal p = 0.02; Fig. 2B), with the greatest change in mucosal-associated microbiota observed in the antibiotic pretreated, capsule-delivered FMT treatment group compared to the non-antibiotic pretreated subjects in the capsule-delivered group (Wilcoxon p = 0.04). Notably, no significant difference was observed in the enema-treated groups based on antimicrobial administration (Wilcoxon p = 1). Thus, these data indicate that a combination of effective antibiotic pre-treatment and capsule-delivered FMT promote the greatest change in transcriptionally active mucosal-associated bacteria in patients undergoing FMT, plausibly by effectively “*priming the distal colon*” for FMT delivery.

Beta-diversity analysis of all subjects (N = 19) across both pre- and post-FMT time points confirmed that the four patient groups exhibited distinct transcriptionally active mucosal-associated microbiota compositions (Axis 3, LME p = 0.04; Fig. 2C). Irrespective of antibiotic pretreatment, mucosal-associated microbiota composition differed between R and NR at both the pre- and post-FMT time points (PERMANOVA p = 0.03 and 0.04, respectively; Fig. S4), suggesting that both the pre- and post-FMT mucosal-associated microbiota relate with clinical response.

We next identified specific mucosal-associated bacteria that related with treatment response by comparing the relative abundances of amplicon sequence variants (ASV) of all NR patients to those of R patients (stratified by time point and antibiotic pretreatment; Fig. S5). Pre-FMT, comparing the NR to the Abx+ R group revealed the greatest number of differentially abundant ASVs (Fig. 2D), the majority of which were significantly increased in the NR group and included members of *Bacteroides* (Figs. 2E; S5A). Notably, this was the only genus that remained differentially enriched on NR mucosa post-FMT when compared to the Abx+ R group (Fig. S5C), indicating that sustained colonization by members of this genus associates with subsequent FMT failure. While the number of subjects is small relative to the number of comparisons performed, these results suggest that mucosal-associated microbiota relate to FMT efficacy. More specifically, they indicate that a combination of antibiotic pretreatment and capsule delivered FMT produces the greatest change in mucosa-associated microbiota and that expression of antimicrobial resistance genes and sustained presence of transcriptionally active *Bacteroides* on the mucosa associate with FMT failure.

### Colonic mucosal-associated innate immune signaling pathways are upregulated in responders

To more broadly understand mucosal-associated microbial transcriptional programs and host responses associated with FMT outcomes, we conducted dual (microbial and human) RNA-sequencing (RNA-seq) on colonic mucosal biopsy samples from NR and R patients (N = 36) at both pre- and post-FMT time points. Using a data reduction approach (Weighted Gene Co-Expression Network Analysis) we identified 15 modules of co-associated human mucosal transcripts (Fig. S6). In the combined analysis of pre- and post-FMT samples, four modules (M1–M4) were significantly associated with FMT response (Fig. S7; LME; FDR-adjusted p < 0.05). R patients exhibited decreased median mucosal expression of modules M1, 2 and 3, which were characterized by transcripts of genes involved in post-translational protein modifications and fructose metabolism (M1), amino acid metabolism, including tryptophan and the branched chain amino acids valine, leucine, isoleucine (M2), and transcription and translation (M3; Table S3). The mucosa of FMT-responsive patients exhibited increased expression of a single module (M4), predominantly characterized by genes involved in cytokine signaling, innate immunity, and responses to viruses and bacteria, including *IL6R* and *IRF7* that aid in mounting antimicrobial and pathogen clearance immune responses^31,32^. These four modules did not significantly differ between antibiotic-pretreated and non-antibiotic pretreated responders (p > 0.05), suggesting that they represent key features of mucosal transcriptional reprogramming that exist pre-FMT and underlie successful treatment. These data also indicate that restoration of innate immune capacity to respond to microbes, in parallel with changes in mucosal-associated transcriptionally active bacteria is critical to FMT-response.

### Fecal microbial species differ between non-responders and responders following cessation of treatment

**Figure 3:**
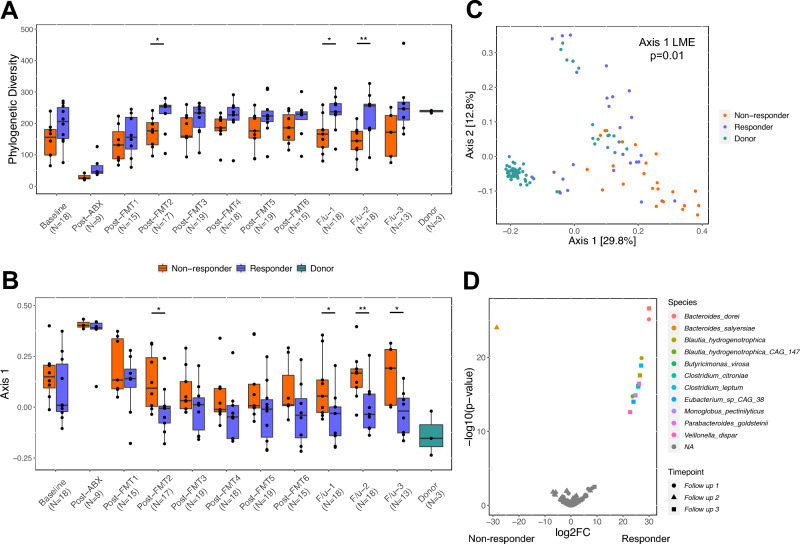
FMT-responsive patients exhibit sustained microbiota differences that are sustained post-treatment. (**A**) Faith’s phylogenetic diversity is displayed for each time point in non-responders (orange), responders (blue), and donors (green). (**B**) Axis 1 (Unweighted UniFrac) is displayed for each time point in non-responders, responders, and donors. Pairwise comparisons between non-responders and responders were performed using a Wilcoxon rank sum test (*p < 0.1; **p < 0.05). For both (**A**) and (**B**), the mean value of Faith’s phylogenetic diversity and Axis 1 was computed for a total of 48 samples from donor 1, 6 samples from donor 2, and 11 samples from donor 3. (**C**) Using patient samples at all follow-up time points and donors, beta diversity (Unweighted UniFrac) was compared between non-responders, responders, and donors using a linear mixed effects model. (**D**) Volcano plot of differentially abundant species (DESeq2; FDR-adjusted p < 0.05); colors indicate species and shapes indicate follow-up time points 1, 2, and 3. “NA” indicates species that were not significantly different. ABX, antibiotic; FMT, fecal microbial transplantation; F/u, follow-up.

Luminal colonic microbiome composition, functional capacity and metabolic productivity is distinct in UC patients and healthy subjects^33,34^. We thus hypothesized that fecal microbiomes differ between R and NR groups and that the former would exhibit restoration of microbial pathways and metabolic products characteristic of healthy donors. Feces of the 19 patients were collected before, during, and after the clinical trial and subjected to 16S rRNA sequencing (n=179 samples). Consistent with previous reports^35,36^, diversity of all UC patient baseline microbiota was modestly decreased compared to healthy donors (Wilcoxon p = 0.12) with a significant difference in phylogenetic diversity observed between NR and donor groups pre-FMT (Wilcoxon p = 0.03; Fig. 3A; Table S4). For those randomized to antibiotic pretreatment groups, antibiotic administration further reduced UC fecal bacterial diversity compared to baseline samples. Subsequent FMT treatments promoted re-diversification, with modest differences detected between R and NR patients at four of six time points during the FMT treatment period (P ≤ 0.1; Table  S4). Following cessation of FMT, compared to NR patients, fecal microbiota diversity of R patients and donors was higher at both the first and second follow-up time points (Wilcoxon p ≤ 0.1). In contrast, the fecal microbiota diversity of R patients did not show significant differences from the donors at any time point throughout the 6-week FMT treatment period and across all three follow-up periods. This indicates that sustained alterations in fecal microbial diversity occurred solely in the FMT-responsive patients, both during and after treatment.

An examination of microbiota beta-diversity revealed similar trends. No difference was observed between NR and R groups at baseline (PERMANOVA R^2^ = 0.05, p = 0.52; Fig. S8A), but significant differences were evident in all three of the post-FMT follow-up sampling period (Fig. 3B; PERMANOVA R^2^ = 0.11, 0.12, and 0.13, respectively and, P = 0.02, 0.007, and 0.03, respectively; Fig. S8B–D), with the R group fecal microbiota more closely resembling that of the donor (Fig. 3C). These observations suggest that successful FMT leads to sustained fecal microbiota compositional changes for several months after cessation of treatment.

To identify the microbial species and functional traits associated with FMT success, we performed shotgun metagenomic sequencing on longitudinal fecal samples, classified species using *HUMAnN 3.0 *^37^ and performed differential abundance analysis. Species that differentiated R and NR groups at the follow-up time points (Fig. 3D) included *Bacteroides dorei*, *Blautia hydrogenotrophica*, and *Blautia hydrogenotrophica CAG 14* that were consistently enriched in the R group at more than one follow-up time point (Fig. S9). Conversely, *B. fragilis, B. fragilis CAG 47 and B. salyersiae* were enriched in the NR group, primarily at the second follow-up sampling point. Where possible, we used *StrainFacts*^38^ to assess the source of these species. We demonstrated that distinct strains of *B. hydrogenotrophica* were present in both the donor material and the fecal microbiomes of R patients and were sustained following cessation of FMT (Fig. S10). In contrast, strains of *B. dorei* were found in all patients with no clear difference in composition between R and NR groups (Fig. S11). These results suggest that engraftment of *B. hydrogenotrophica* may contribute to the efficacy of FMT.

### Fecal metabolic sub-pathways relate to treatment response

**Figure 4:**
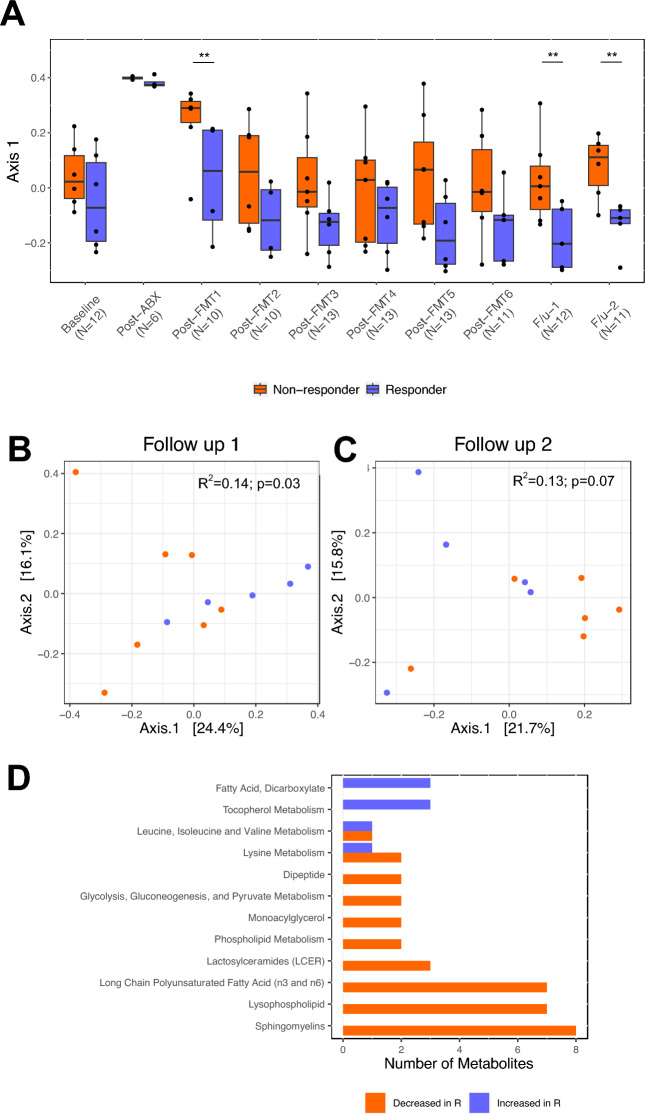
Distinct fecal metabolic sub pathways associate with FMT responsiveness. (**A**) Axis 1 (Bray–Curtis) is displayed for each time point in non-responders (orange) and responders (blue). Pairwise comparisons were performed using a Wilcoxon rank sum test (**p < 0.05). Stool samples collected at follow-up 3 (F/u-3) did not undergo parallel metabolomics profiling. Beta diversity (Bray–Curtis) was compared using PERMANOVA between non-responders and responders at the (**B**) first and (**C**) second follow-up time points. (**D**) For 56 differentially abundant metabolites at the first follow-up time point (DESeq2; FDR-adjusted p < 0.05), metabolic sub pathways that included two or more metabolites are colored by the number of metabolites increased in responders (blue) and decreased in responders (orange). R; responder.

We next profiled the fecal metabolome using untargeted metabolomics. Consistent with our earlier observations, no difference was detected in the baseline fecal metabolomes of R and NR patients (PERMANOVA R^2^ = 0.09, p = 0.41, Fig. 4A). However, a significant difference in fecal metabolomes was evident at the first follow-up time point (PERMANOVA R^2^ = 0.14; p = 0.03; Fig. 4B) and trended at the second follow-up time point (PERMANOVA R^2^ = 0.13; p = 0.07, respectively; Fig. 4C). At each of these time points, axis 1 of the PCoA plot differentiated R and NR groups. Therefore, to identify metabolites associated with FMT responsiveness we tested correlations with this axis and identified 56 and 9 response-associated metabolites at the first and second follow-up time points, respectively (Spearman; FDR p < 0.05; Fig. S12A,B). Response-associated metabolites at the first follow-up time point included sphingomyelins, lysophospholipids (e.g. 1-stearoyl-glycerophosphoglycerol and 1-stearoyl-glycerophosphocholine), and long chain polyunsaturated fatty acids including docosapentaenoate, dihomolinolenate and arachidonate that were notably depleted in the R group (Fig. 4D). Metabolites increased in the R group included the dicarboxylic fatty acids pipecolate, glutarate and several vitamin E (tocopherol) metabolites. At the second follow-up time point, pipecholate remained enriched in FMT-responsive patients who were also characterized by depletion of derivatives of tyrosine, ornithine and taurine amongst others. Using metabolite enrichment analysis (MetaboAnalyst) we noted that the metabolites found to differentiate R and NR groups at both follow-up time points mapped to diseases that included ulcerative colitis, Crohn’s disease, and other immune-mediated diseases (Fig. S12C,D), providing further support that these metabolites are involved in ulcerative colitis pathogenesis.

### Microbiome-derived metabolites associate with treatment response and disease severity

**Figure 5:**
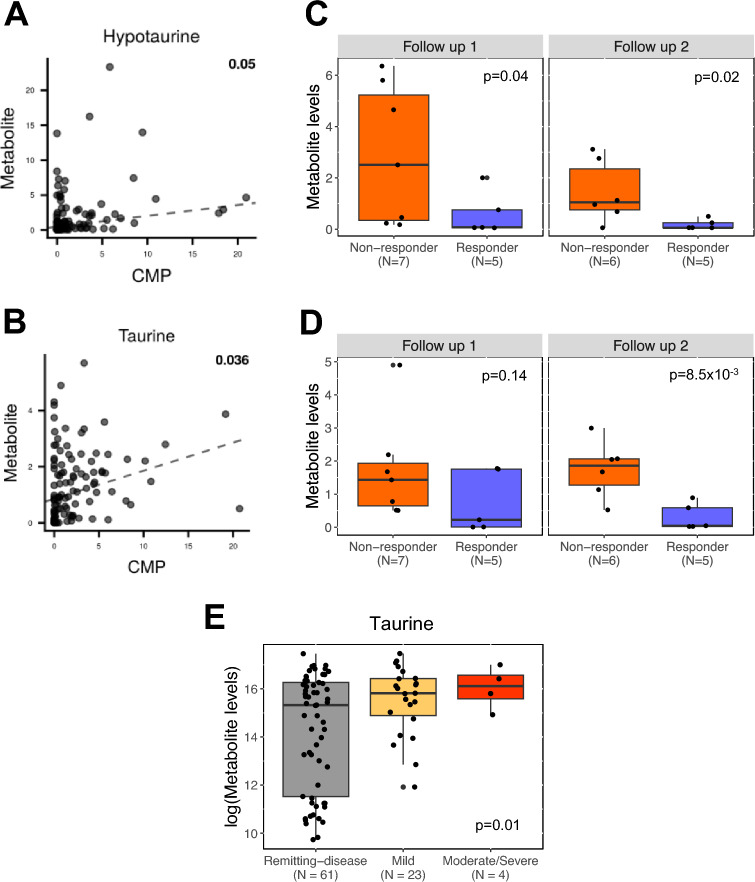
Loss of microbes with the capacity to produce hypotaurine and taurine associates with FMT responsiveness. Integration of fecal shotgun metagenomic (*HumaNn3*) and metabolomic datasets (Metabolon) was performed using *MIMOSA2* and identified (**A**) hypotaurine and (**B**) taurine as fecal microbiome-derived metabolites. Microbial producers of both metabolites are shown in Table S6. (**C**,**D**) Normalized abundance levels for each metabolite are displayed for non-responders and responders at the first and second follow-up time points. Comparisons between non-responders and responders were performed using a one-sided Wilcoxon rank-sum test. (**E**) From the IBDMD cohort, log-transformed abundance levels for taurine are shown by ulcerative colitis severity groups, as defined by SCCAI categories. An association between metabolite abundance levels and SCCAI categories were tested using an ordinal logistic regression. See Table S6 for the microbial species and their genes that metabolize hypotaurine and taurine. CMP; community metabolic potential score.

Both microbial and metabolomic profiles differed between R and NR groups at the follow-up time points, suggesting that microbiome-derived metabolites relate to FMT outcomes. To link microbial species and genes with metabolites found to be related to FMT response, paired shotgun metagenomic and metabolomic data (n = 110) were analyzed with *MIMOSA2*, an integrative, metabolic model-based analysis tool^39^*.* Specifically, response-associated bacterial species and all metabolites were used as input for *MIMOSA2* to identify microbiome-derived metabolites that relate to FMT efficacy. Post-FMT, bacterial species found to be relatively increased in R patients (Fig. S9A,C) were significantly associated with fecal concentrations of two metabolites, l-arginine and 4-coumarate (FDR p < 0.05; Table S5; Fig. S13); four species (*B. dorei*, two strains of *B. hydrogenotrophica*, and *B. virosa*) encoded two different enzymes that metabolize l-arginine. Consistent with these results, l-arginine levels were significantly reduced at the second follow-up time point (One-sided Wilcoxon p = 0.02). Both strains of *B. hydrogenotrophica* also metabolized 4-coumarate although the metabolite levels were not significantly different between the R and NR groups at follow-up 2 (One-sided Wilcoxon p > 0.05). Bacterial species found to be relatively increased in NR patients (*B. fragilis, B. fragilis CAG 47 and B. salyersiae*; Fig. S9B) were significantly associated with fecal concentrations of four metabolites—hypotaurine, taurine, choline, and l-alanine (FDR p < 0.05; Fig. 5A,B; Table S6; Fig. S14). Both *Bacteroides fragilis* strains encoded genes with the capacity to produce all four metabolites, while *B. salyersiae* encoded the same enzymes as both *B. fragilis* strains that produce hypotaurine and taurine (Table S6). Consistent with these results, hypotaurine and taurine levels were significantly elevated in NR compared with R patients at the second follow-up time point (One-sided Wilcoxon p < 0.05; Fig. 5C,D). To validate these findings, we examined metabolomic data from the Inflammatory Bowel Disease Multi’omics Database (IBDMD) Project^40^ and found that taurine, as well as l-alanine and choline concentrations significantly associated with the severity of ulcerative colitis (defined by SCCAI severity categories^41^; ordinal logistic regression p < 0.05; Figs. 5E; S15). These data reveal microbial-derived fecal metabolites that relate to clinical response to FMT and to severity of UC.

## Discussion

The success of fecal microbial transplantation (FMT) in clinical studies varies considerably, likely due to factors such as differing clinical protocols for FMT preparation (i.e. antibiotics, bowel lavage) and administration (i.e. capsule, enema, nasoduodenal or colonic delivery and frequency), as well as microbiological and host factors^42,43^. In this study, we investigated microbiological factors associated with FMT clinical response by profiling both the colonic mucosal and luminal compartments. Our results revealed that antibiotic pretreatment and serial FMT delivery via capsules led to the greatest change in the mucosal-associated microbiota post-FMT cessation. Furthermore, reduced expression of antimicrobial resistance determinants by mucosal-associated microbes following antimicrobial pre-treatment was characteristic of those who responded to treatment. This finding is consistent with previous studies that reported a significant association between antibiotic resistance and FMT-non-responsiveness in UC patients^44^, suggesting that failure to eradicate antibiotic-resistant bacterial strains priot to FMT associates with treatment-failure.

Prior to treatment, no significant difference was observed between responders and non-responders in fecal microbiota composition, functional capacity, or metabolome profiles. However, several fecal bacterial species were found to be related to treatment response and sustained post-FMT. We demonstrate that *B. fragilis* and *B. salyersiae* were significantly enriched in the lumen of non-responsive patients post-FMT. *Bacteroides* species are among the most commonly abundant species of the gut and typically reside in the outer mucosal layer of the colon^45^. Interestingly, these species have been associated with pro-inflammatory properties and invasive behavior, allowing for colonization of intestinal epithelia^46^. A previous study from our group identified a relative expansion of *Bacteroides* in UC patients that exhibited the most severe disease^10^. This finding has been replicated in independent UC microbiome studies^9,47^. Moreover, *Bacteroides* enterotoxigenic proteases have been correlated with the onset of UC^33,48^ and IBD^49^. *B. fragilis* was also found to induce colitis in murine models^50^. Our data indicating sustained colonic mucosal presence of *Bacteroides* species, particularly *B. fragilis* in NR patients, aligns with previous findings showing that 5-aminosalicylic acid and antibiotics, suppress but do not eliminate colonic-adherent *B. fragilis* biofilms in IBD patients^51^. Collectively, these observations highlight the potential role of *Bacteroides* species, especially persistent pathogenic *B. fragilis* and *B. salyersiae* in UC pathogenesis and their association with FMT treatment failure and suggest they may represent target species, whose depletion may reduce inflammation and improve FMT outcomes in UC patients.

Using an integrative metagenomics-metabolomics approach, we linked clinical response-associated microbial genes to metabolites that differentiate R and NR patients. *B. fragilis* encoded gene products that produced all four metabolites (hypotaurine, taurine, choline and l-alanine) associated with the NR group. Both *B. fragilis* and *B. salyersiae* encoded hypotaurine and taurine metabolizing genes and were associated with an increased abundance of both of these metabolites. Although taurine has been found to reduce inflammation in experimental murine models of IBD^52,53^, most studies in patients with UC and Crohn’s disease have reported increased taurine metabolism^54,55^. Taurine has a role in modulating NLRP6 inflammasome signaling in the intestine^56^ and it has also been found to modulate epithelial IL-18 secretion and downstream antimicrobial peptide production, key processes in maintaining intestinal homeostasis^56^. Our findings suggest that *B. fragilis* and *B. salyersiae*, which encoded genes responsible for metabolizing hypotaurine and taurine, may contribute to inflammation and colonic mucosal damage associated with UC by regulating the pool of available taurine.

Using an independent cohort^40^, we also found that levels of the *B. fragilis* and *B. salyersiae*-produced metabolites, taurine, l-alanine, and choline, were associated with more severe cases of UC. Choline is an essential nutrient acquired through the diet, and gut microbes hydrolyze phosphatidylcholine to release choline for downstream metabolism^57^. Trimethylamine, produced by gut microbes from choline^58^, has been implicated in various adverse host pathologies, including IBD^59^. Inhibiting microbes capable of producing choline could potentially represent a therapeutic approach for treating trimethylamine-induced pathologies. Taken altogether, these findings suggest that these microbially-derived metabolites may not only relate to FMT failure but also contribute to the inflammation and colonic mucosal damage that is associated with severe cases of UC.

In contrast, FMT-responsive patients exhibited increased abundance of *Blautia hydrogenotrophica*, *Bacteroides dorei*, and *Butyricimonas virosa* post-FMT. Notably, these bacteria were found to encode two arginine metabolizing genes, which were associated with decreased abundance of l-arginine in these patients. l-arginine is a semi-essential amino acid, and a central gut metabolite. It is converted by host macrophages into ornithine, urea, nitric oxide, and citrulline, some of which have key functions in maintaining cellular homeostasis and in regulating cell proliferation, tissue repair, and wound healing^60^. Competition for l-arginine among bacteria, immune cells, and the host can also influence immune cell function and colitis development^61^. Modulating l-arginine availability and gut microbial metabolism may have therapeutic implications for immune system modulation and UC treatment.

In addition to the microbial factors, we found that FMT-responsive patients exhibited increased transcription of innate immune signaling pathways in the colonic mucosa. Increased mucosal expression of a module of innate immune genes, including *IL6R* and *IRF7*, was associated with successful FMT. This finding suggests that effective bacterial burden reduction via antimicrobials coupled with restored capacity to mount an effective antimicrobial response at the mucosal surface and maintain a pathogen-free intestinal mucosa is characteristic of treatment-responsive patients^31,32^.

Several limitations of this study merit consideration, the most important of which stems from enrollment and attrition. The four-arm trial design, initially intended to explore the optimal approach (pre-conditioning with antibiotics, and capsule versus enema), was affected by the unpredictable global crisis of SARS-Cov-2, which hindered our ability to recruit and retain the participants required to answer these important clinical questions. Initially, we planned to enroll 40 patients and randomly assign them to four treatment arms, but ultimately only 19 participants completed the study. Additionally, this open-label trial lacked a placebo arm; without this, the efficacy directly attributable to the treatments could not be ascertained. Third, given the exploratory nature of this study, we targeted enrollment of patients with mild-moderate UC and excluded those with severe disease who might have responded differently to the treatments. Despite these challenges, we were able to successfully identify known immunomodulatory metabolites that exhibited significant differences between healthy individuals and those with UC in previous studies^54,55^. Additionally, the inclusion of repeated measures throughout the duration of the study has further enhanced our ability to detect microbiological differentials between R and NR patients.

Altogether, we identified gut microbial species, particularly *B. fragilis* and *B. salyersiae*, that associate with FMT non-responsiveness and potentially contribute to UC pathogenesis. Furthermore, we identified microbiome-derived metabolites, such as taurine and hypotaurine, that are associated with inflammation and failed microbial clearance. These metabolites may hinder the success of FMT and contribute to the severity of UC. Targeting these microbial species and their associated metabolites could offer a promising approach for clinical intervention in UC patients. Further studies are needed to evaluate the potential of these interventions in modulating the immune system and achieving UC remission.

## Materials and methods

### Study participants and randomization

An open-labelled prospective study of adult ulcerative colitis patents between 18 and 64 years old was performed at the Colitis and Crohn’s Center of the University of California, San Francisco (UCSF) from September, 2017 to May, 2019 (clinicaltrials.gov NCT03006809)^13^. All methods were carried out in accordance with relevant guidelines and regulations of the UCSF. All participants provided written informed consent. The study was approved by the Institutional Review Board of the Human Research Protection Program of the UCSF.

Participants were recruited after evaluation in gastroenterology clinic followed by a flexible sigmoidoscopy if no endoscopic evaluation had been completed within one year of enrollment. Patients assessed to have mild-moderate UC (Mayo Score 4–9) were enrolled. Stable medications were allowed to continue; steroids were weaned following enrollment. See Supplementary Methods for a list of all inclusion and exclusion criteria. Upon enrollment, participants were randomized to one of four arms, which differed by route of delivery of consecutive FMT (capsule versus enema) and presence or absence of twice daily pretreatment with antibiotic cocktail (vancomycin 500 mg, metronidazole 500 mg, neomycin 500 mg) for five days prior to the initial colonoscopy.

### Fecal microbial transplantation and clinical endpoints

All participants prepared for the initial colonoscopy using a standard bowel lavage solution of polyethylene glycol. Pre-screened donor FMT was prepared by a stool bank (OpenBiome, MA), the screening process of which has been described previously^62^. Participants underwent colonoscopy with rectal biopsy, and prepared FMT (250 mL) was initially colonoscopically administered into the terminal ileum. Beginning 1 week after initial colonoscopy, each participant underwent six rounds of consecutive weekly FMT, administered either via the ingestion of 30 capsules or rectal delivery of 60 mL enema, both of which were prepared by the same stool bank. Each participant received fecal material from the same donor (of three) throughout the entirety of the study. Two weeks after the sixth dose of capsules or enema, participants underwent repeat colonoscopy with rectal biopsy. All rectal biopsies obtained were immediately preserved in RNAlater according to the manufacturer’s instructions (Thermo Fisher Scientific, MA) and stored at − 80 °C for subsequent nucleic acid extractions or in 10% buffered formalin for histologic evaluation of haematoxlyin and eosin-stained sections.

Disease severity of each participant was assessed using the full Mayo Score at two predefined intervals. The first Mayo Score was determined upon enrollment and completed by supplementation of endoscopic score from the initial colonoscopy. The second Mayo Score, used to determine treatment response, was determined at the time of the second colonoscopy.

### Rectal biopsy nucleic acid extraction and processing

DNA and total RNA were extracted and purified in parallel from mucosal biopsies using the ZymoBIOMICS DNA/RNA Mini-Prep kit (Zymo Research, CA) following manufacturer’s protocol with the following adjustment: bead-beating was performed for 30 s at 5.5 m/s. DNA was removed from RNA preparations using DNase I (Turbo DNase, Thermo Fisher Scientific, MA). RNA extracts were confirmed DNA-free by using 5 µL RNA extract as template in a single 16S rRNA gene PCR followed by gel electrophoresis (presence of a band indicated DNA-contamination). RNA was quantified using Qubit RNA HS assay (Thermo Fisher), normalized, and converted to single-stranded cDNA (High-Capacity RNA-to-cDNA Kit, Thermo Fisher) in a 20 µL reaction.

### Stool nucleic acid extraction and processing

Collection and processing of stool samples for microbiome analyses are described previously^13^. In brief, stool samples were collected by patients at home using a custom collection kit and shipped overnight to the investigators. Upon receipt, samples were stored at − 80 °C. Collection intervals were determined during drafting of the protocol prior to enrollment of the first patient described in Supplementary Methods. When available, aliquots of donor samples were also collected and stored at − 20 °C until processed for DNA extraction.

Extraction of DNA from fecal samples was performed using the modified cetyltrimethylammonium bromide (CTAB) as previously described^63^. In brief, an aliquot (~ 0.3 g) was taken from each frozen stool sample and suspended in 500 µL CTAB extraction buffer in a Lysing Matrix E tube (MP Biomedicals) by vortexing and subsequently incubating for 15 min at 65 °C. After adding 500 µL phenol:chloroform:isoamyl alcohol (25:24:1), the solution underwent bead-beating (5.5 m/s for 30 s), followed by centrifugation (16,000*g* for 5 min at 4 °C). The resulting aqueous phase (approximately 400 µL) was transferred to a new 2 mL 96-well plate (USA Scientific). An additional 500 µL CTAB extraction buffer was added to the fecal aliquot and subsequent steps were repeated, resulting in approximately 800 µL from repeated extractions. Equal volume of chloroform was added and the solution mixed and centrifuged (3000*g* for 10 min). The resulting aqueous phase (approximately 600 µl) was transferred to another 2 mL 96-well plate (USA Scientific), combined with 2-volume polyethylene glycol (PEG) and stored at 4 °C overnight to precipitate DNA. Samples were then centrifuged (3000*g* for 60 min), washed twice with ice cold 70% EtOH, and resuspended in sterile water and diluted to 10 ng/µL (Qubit dsDNA BR Assay Kit; ThermoFisher Scientific, MA).

### Amplicon library preparation, sequencing and data processing

The V4 region of the 16S rRNA gene was amplified via PCR using primers and conditions previously described^63^ using either DNA (fecal samples) or cDNA (mucosal samples) as template. PCR reactions were performed with 0.625 U Hot Start ExTaq (Takara Bio USA, CA) and 1× buffer, 200 µM dNTPs, 0.56 µL/µL BSA (Roche), 0.4 µM each forward (F515) and reverse (R806) primers in triplicates of 25 µL reactions containing 10 ng of template nucleic acid (DNA for stool, cDNA for mucosal biopsies). Thermal cycling was set at: 98 °C for 2 min, 30 rounds of 98 °C 20 s, 50 °C 30 s, 72 °C 45 s, and a final extension at 72 °C for 10 min. Amplicons were normalized (SequalPrep Normalization Plate Kit; ThermoFisher Scientific, MA), quantified (Qubit dsDNA BR Assay Kit; ThermoFisher Scientific, MA), pooled in equimolar concentrations, purified (Agencourt AMPure XP System; Beckman-Coulter), and quantified (KAPA Library Quantification Kit; KAPA Biosystems). Fecal amplicons were diluted to 2 nM, Equimolar PhiX spike-in control was added at 40% final volume, and the samples were sequenced on Illumina NextSeq 500 Platform producing paired end 300 × 300 bp reads. Mucosal amplicons were diluted to 1.5 nM, PhiX spike-in control was added at 27% final concentration, and the samples were sequenced on Illumina MiSeq Platform producing paired end 300 × 300 bp reads.

BCL files were converted to fastq using Illumina’s bcl2fastq software v2.20.0.422. Reads were demultiplexed by barcode using QIIME^64^. Pre-processing (filtering, trimming, dereplication, merging of paired reads, removal of primers and chimeras) and assigning of taxonomy against the Silva v138 database were carried out using the DADA2 package^65^. A phylogenetic tree was constructed using the phangorn and DECIPHER packages^66^. ASVs with < 0.0001% total read count across all samples were removed. For the fecal ASVs, negative control filtering was performed by removing ASVs that were present in > 15% of negative controls and in < 15% of samples. For the mucosal-associated ASVs, more stringent negative control filtering was performed to account for the low burden of bacteria expected in this sample type; ASVs were removed if present in > 50% of negative controls and whose proportion of reads across all negative controls was greater than proportion of reads across samples. Among remaining ASVs, the average read count within negative controls was subtracted from the read count in samples. To normalize variation in read-depth across samples, data underwent variance stabilized transformation using DESeq2^67^. The normalized ASV table was imported into the phyloseq R package for further analysis^68^.

### Dual RNA-seq library preparation, sequencing and data processing

Quality of DNA-free RNA was determined by Bioanalyzer (Agilent). Total RNA was converted to double-stranded cDNA, fragmented, and ligated with Illumina adapters using the NEBNext Ultra II Directional RNA Library Prep Kit for Illumina (New England BioLabs, MA), following protocol for partially degraded RNA (RIN 2–7) or intact RNA (RIN > 7) as determined by BioAnalyzer tracings. RNA was sequenced on a NovaSeq6000 sequencer using one lane, producing paired-end 150 × 150 bp reads.

Reads were demultiplexed, quality filtered, and removed of Illumina adapters using BBDuk^69^. Quality control checks were performed using FastQC. Reads were aligned to the human genome (Hg38 release) and features were assigned to transcripts using Spliced Transcripts Alignment to a Reference (STAR)^70^. Genes with low count data (< 10 counts per million in at least 10% of the samples) and those on the X, Y, and mitochondrial chromosomes were removed. Raw human mRNA counts were normalized using the trimmed mean of M-values (TMM) method^71^. Mean–variance trend was adjusted using variance modeling in voom^72^. Technical sources of variation were identified using principal components analysis (PCA) and a linear mixed effects model. Processed genes were clustered into co-expression modules using WGCNA^73^ and genes within each module were subjected to pathway analysis using TopFunn^74^. For all enriched pathways, each individual was assigned a pathway score based on the median expression of all input genes.

Reads that did not map to the human genome were subjected to the IDSeq automated pipeline^30^, which performed QC, alignments to microbial genomes, and screened for the presence of antibiotic resistance determinants.

### 16S copy number qPCR

16S rRNA gene copy number was assessed by quantitative PCR (qPCR) using the 16S rRNA universal primers and TaqMan probes, as previously described^75^. Briefly, total 16S rRNA gene copy number was calculated against a standard curve of known 16S rRNA copy numbers (1 × 10^2^–1 × 10^9^). The qPCR was performed in triplicate 20-µl reactions containing final concentrations of 1 × TaqMan Universal Master Mix (Life Technologies), 1 ng of cDNA produced from RNA-seq library preparation, 900 nM of each primer, P891F (5′-seq-3′F) and P1033R (5′-seq-3′R) and 125 nM of UniProbe under the following conditions: 50 °C for 2 min, 95 °C for 10 min, followed by 40 cycles of denaturation at 95 °C for 15 s and annealing and extension at 60 °C for 1 min, along with a no-template control and eight standards. For each patient, the mean of the 16S rRNA copy number across replicates was used for downstream analyses.

### Shotgun metagenomic sequencing and processing

DNA was extracted from patient and donor stool samples using the modified CTAB methods, and sequenced by QB3 at the University of California, Berkeley for 150-bp paired-end sequencing on the NovaSeq 600 S4 platform (https://qb3.berkeley.edu/). Raw FASTQ files underwent quality control (FASTQC) and quality and contaminant filtering using bbTools v38.73^69^, as previously described^76^. Briefly, bbduk removed PhiX contamination and trimmed Illumina adapters and low-quality sequence and bbmap removed reads mapping to the human genome (GRCh38^77^). HUMAnN 3.0 was used to identify functional features of the fecal microbiomes. UniRef labels were assigned, data was normalized to copies per million and UniRef90 IDs were converted into MetaCyc reactions. MetaCyc reactions that were present in < 20% of samples were removed and samples with < 20% of detectable MetaCyc reactions were removed, yielding 608 MetaCyc reactions and 218 fecal microbiome samples for downstream analyses.

### Metabolomics profiling and processing

Parallel metabolomics profiling for fecal samples (200 mg) was provided to Metabolon (Durham, NC) for Ultrahigh Performance Liquid Chromotography/Tandem Mass Spectrometry (UPLC-MS/MS) and Gas Chromatography-Mass Spectrometry (GC–MS) using their standardized protocol (http://www.metabolon.com/). Detected molecules were identified through Metabolon’s in-house library of purified standards encompassing more than 3300 commercially available compounds. This yielded 1050 distinct metabolites, from which those present in < 20% of all samples (426 metabolites) were removed from the metabolite table. Detection values of the remaining 624 metabolites were normalized by extracted mass. For samples in which certain metabolites were not detected, the minimum detection value across all samples for that metabolite was imputed. All metabolite values were subsequently log-transformed and auto scaled.

To validate our metabolic findings, we leveraged data from The Inflammatory Bowel Disease Multi’omics Database Project (IBDMD), an integrated resource that used a cohort from the Integrative Human Microbiome Project (HMP2) to longitudinally characterize host and microbial profiles during disease^40^. We extracted the fecal metabolite abundance levels of the microbiome-derived metabolites, as identified by *MIMOSA2*. Of four LC–MS methods (HILIC-pos, HILIC-neg, C18-neg, and C8-pos) used to profile metabolites, we collected data for the method (HILIC-pos) that profiled the most metabolites of interest (taurine, l-alanine, choline, and l-arginine). Hypotaurine and 4-coumarate were not detected using any of the four LC–MS methods. In addition to metabolite abundance levels, the simple clinical colitis activity index (SCCAI) was obtained and categorized into SCCAI severity groups (remitting-disease, SCCAI = 1,2; mild, SCCAI = 3,4,5; moderate/severe, SCCAI > 5); patients with moderate and severe disease were combined to increase statistical power^41^

### Statistical analyses

All statistical analyses were completed in R v.4.1.2. Mucosal-associated 16S rRNA copy number and normalized read counts were compared between antibiotic response groups (antibiotic pretreated non-responders, non-antibiotic pretreated non-responders, antibiotic pretreated responders, and non-antibiotic pretreated responders) in the pre-FMT and post-FMT samples, separately, using a Kruskal–Wallis test. To assess bacterial change after FMT, the difference in 16S rRNA copy number was calculated between pre- and post-FMT samples. Both the bacterial change after FMT and the number of AMR genes, as detected by IDseq, were compared between non-responders and responders using a Wilcoxon rank sum test.

Variables that were associated with shifts in the mucosal-associated transcriptionally active organisms after FMT were identified by calculating paired unweighted Unifrac distances (*distance* function from phyloseq v1.38.0) between pre-FMT and post-FMT samples for each patient and comparing distances between groups using either a Wilcoxon rank sum test or a Kruskal–Wallis test. The paired Unifrac distances were not associated with any potential biological confounders including sex, age, biologic therapy use, steroid therapy use, years since diagnosis, or extent of disease (P > 0.05), thus the tests of associations with clinical variables including response, antibiotic use, or mode of FMT delivery did not include covariates. We next ordinated the unweighted Unifrac distance matrix into three-dimensional space using the *pcoa* function from the ape v5.6 package and tested the association of the first three axes and antibiotic response including both pre-FMT and post-FMT samples using a linear mixed effects model to account for the effect of patient. At each time point, permutational analysis of variance tests (PERMANOVA; adonis2 from the vegan package v2.6-2) was performed to compare non-responders and responders. Differential abundance analyses of individual ASVs between responders and non-responders were performed using DESeq2 v1.34.0 and response-associated ASVs were detected at FDR < 0.05.

Using the fecal 16S rRNA data, Faith’s phylogenetic diversity metric was compared between non-responders and responders at all time points with a Wilcoxon rank sum test. An unweighted Unifrac distance matrix was constructed and permutational analysis of variance tests (PERMANOVA; adonis2 from the vegan package v2.6-2) performed to test associations of fecal 16S rRNA profiles with all measured variables. Fecal 16S rRNA composition was not associated with any potential biological confounders including sex, age, biologic therapy use, steroid therapy use, years since diagnosis, or extent of disease or with antibiotic administration or mode of FMT delivery (P > 0.05). However, the fecal 16S rRNA profiles associated with response, specifically at the follow-up time points. Thus, the focus of downstream analyses was on the follow-up time points.

Similar methods were applied to the fecal metagenomics (microbial species) and the metabolomics data. For both datasets, a distance matrix (Unweighted UniFrac for microbial species-level data and Bray–Curtis for metabolomics abundance data) was generated and the first axis was used in downstream analyses. PERMANOVA was performed on the distance matrix to compare non-responders and responders. To identify response-associated microbial species and metabolites at each follow-up time point, the first axis was tested for correlations (Spearman) with all microbial species or all metabolites. For the microbial species-level data only, differential abundance analyses of all microbial species were performed between non-responders and responders using DESeq2 v1.34.0. Using either approach, response-associated microbial species and metabolites were detected at FDR < 0.05. Significant metabolites were further characterized by sub pathways, as defined by Metabolon, and were tested for disease-enriched metabolite sets using MetaboAnalyst.

Integrative analyses between fecal metagenomics (microbial species and functions) and metabolomics datasets were performed using the web application, *MIMOSA2 *^39^. Briefly, this method relates variation in microbiome data to paired metabolite abundances, using information from reference databases on the metabolic capabilities of microbial species, to identify microbiome-derived metabolites (FDR < 0.05) and their linked microbial species and genes. We performed two analyses: (1) including eight microbial species (and their functions) that were increased in responders and (2) including three microbial species (and their functions) that were increased in non-responders. Samples from the entire trial and all metabolites that were detected in > 20% of samples were supplied for both *MIMOSA2* analyses. The microbiome-derived metabolites were compared between non-responders and responders at both follow-up time points using Wilcoxon rank sum tests. Using the independent validation cohort (IBDMD), microbiome-derived metabolites were additionally tested for associations with SCCAI severity categories using an ordinal logistic regression.

### Supplementary Information

Supplementary Information.

## Data Availability

All sequence data will be available on the European Nucleotide Archive at the time of publication. The datasets generated and/or analysed during the current study are available in the short read archive repository under project: PRJNA737472. Additional information available from corresponding author upon request.
