# Supplementary material for: Sustained mucosal colonization and fecal metabolic dysfunction by Bacteroides associates with fecal microbial transplant failure in ulcerative colitis patients
Source: Sci Rep. 2024 Aug 9;14:18558. doi: 10.1038/s41598-024-62463-8 (PMC11316000; doi:10.1038/s41598-024-62463-8)
Supplement: Supplementary file 1 — Supplementary Information. [file 41598_2024_62463_MOESM1_ESM.docx]

**Sustained mucosal colonization and fecal metabolic dysfunction by *Bacteroides* associates with fecal microbial transplant failure in ulcerative colitis patients.**

Bing Zhang^1*^, Kevin M. Magnaye^2,3*^, Emily Stryker^2^, Jacqueline Moltzau Anderson^2,3^, Cara E. Porsche^2^, Sandra Hertz^4^, Kathryn E. McCauley^2^, Byron J. Smith^5^, Martin Zydek^2^, Katherine S. Pollard^5,6,7^, Averil Ma^2^, Najwa El-Nachef^2,8^, Susan V. Lynch^2,3^^

Supplementary Methods

*Inclusion Criteria:*

1. Mild-moderate UC (total Mayo 4-9) confirmed by endoscopy and pathology
2. Patients who have not had endoscopic evaluation within one year of enrollment will have flexible sigmoidoscopy for evaluation
3. Age 18-64 and deemed otherwise healthy at the discretion of the investigator
4. Concurrent therapies with mesalamine, immunomodulators, and biologic agents will be allowed to continue during the study as long as they are on a stable dose for 4 weeks (mesalamine) or 3 months (immunomodulators, biologics)
5. If prednisone is taken, it must be < 10mg/day at the time of treatment and will be weaned by 2.5mg/week during the study period

*Exclusion Criteria:*

1. Severe or refractory UC defined as Mayo ≥ 10
2. Untreated enteric infections
3. History of colectomy
4. Disease limited to distal proctitis
5. Use of probiotic within 6 weeks of planned FMT therapy
6. Severe immunodeficiency, inherited or acquired (ex: HIV, chemotherapy or radiation therapy)
7. Patients with absolute neutrophil count (ANC) < 1000 µL, platelet < 50x10^9^/L, hemoglobin < 6.5 g/dL
8. History of anaphylaxis to food allergens (ex: tree nuts, shellfish)
9. Dysphagia (oropharyngeal, esophageal, functional, neuromuscular)
10. History of recurrent aspiration episodes
11. Documented severe gastroparesis
12. Active intestinal obstruction
13. Renal insufficiency (GFR < 50 mL/min)
14. Allergy to the following generally regarded as safe ingredients (GRAS): glycerol, acid resistant HPMC, gellan gum, cocoa butter, titanium dioxide
15. Adverse event attributable to previous FMT
16. Allergy/intolerance to proton pump inhibitor therapy
17. Allergy/intolerance to vancomycin, metronidazole, or neomycin
18. Non-steroidal anti-inflammatory medications (NSAIDs) as long-term treatment, defined as use for at least 4 days a week each month
19. Cholestyramine use
20. Any conditions in which the investigator thinks the FMT treatment may pose a health risk (ex: severe immunocompromised)
21. Simultaneous participation in another interventional clinical trial
22. Patients who are pregnant, breast feeding or planning pregnancy during study trial period
23. During the trial period until one week after the trial end: Non-use of appropriate contraceptives in females of childbearing potential (ex: condoms, intrauterine device (IUD), hormonal contraception, or other means considered adequate by the responsible investigator) or in males with a child-fathering potential (condoms, or other means considered adequate by the responsible investigator during treatment
24. Well-founded doubt about the patient's cooperation
25. Patients with any other significant medical condition that could confound or interfere with evaluation of safety, tolerability or prevent compliance with the study protocol at the discretion of the investigator
26. Life expectancy < 6 months

Supplementary Tables and Figures

**Table S1. Summary of patient enrollment and analysis inclusion.** Nineteen patients who completed the clinical trial were categorized into one of four arms: antibiotic-pretreated with capsule delivery (Abx+ Caps); non-antibiotic-pretreated with capsule delivery (Abx- Caps); antibiotic-pretreated with enema delivery (Abx+ Enema); non-antibiotic-pretreated with enema delivery (Abx- Enema). Each patient's inclusion in the final analysis for: (**A**) 16S rRNA, (**B**) 16S DNA, (**C**) Metagenomics, (**D**) Metabolomics, and (**E**) Dual RNA-seq is annotated accordingly.

|  |  | **Mucosal** | | **Fecal** | | | | | | | | | | |
| --- | --- | --- | --- | --- | --- | --- | --- | --- | --- | --- | --- | --- | --- | --- |
| **Arm** | **ID** | **Pre-FMT** | **Post-FMT** | **Baseline** | **Post-ABX** | **Post-FMT1** | **Post-FMT2** | **Post-FMT3** | **Post-FMT4** | **Post-FMT5** | **Post-FMT6** | **F/u-1** | **F/u-2** | **F/u-3** |
| Abx+ Caps | DFS.027 | BE | BE | ACD | ACD |  | ACD | ACD | ACD | ACD | ACD | ACD | ACD | AC |
|  | DFS.047 | BE | BE | ACD | ACD | ACD |  | ACD | ACD | ACD |  | AC | AC |  |
|  | DFS.053 |  | BE | ACD | ACD | AD | ACD | ACD | ACD | ACD | ACD | D | ACD | AC |
|  | DFS.063 | BE | BE | AC | AC | AC | AC | AC | AC | AC | AC | AC | AC | AC |
| Abx- Caps | DFS.007 | BE | BE | ACD |  |  |  | ACD | ACD | ACD | ACD | ACD | ACD | AC |
|  | DFS.008 | BE | BE | ACD |  |  | ACD | ACD | ACD | ACD | ACD | ACD | ACD | AC |
|  | DFS.041 | BE | BE |  |  | ACD | ACD | ACD | ACD | ACD | ACD | ACD | ACD |  |
|  | DFS.055 | BE | BE | ACD |  | ACD | ACD | ACD | ACD | ACD | ACD | ACD | ACD | AC |
|  | DFS.059 | BE | BE | AC |  | AC | AC | AC |  | AC |  | AC |  |  |
| Abx+ Enema | DFS.004 | BE | BE | ACD | ACD | ACD | ACD | ACD | ACD | AD | ACD | ACD | ACD | AC |
|  | DFS.021 | BE | BE | ACD | ACD | ACD | ACD | ACD | ACD | ACD |  | ACD | ACD |  |
|  | DFS.024 | BE | BE | ACD | ACD | ACD | ACD | ACD | ACD | ACD | ACD | ACD | ACD | AC |
|  | DFS.060 | BE | BE | AC | AC | AC | AC | AC | AC | AC |  | AC | AC |  |
|  | DFS.064 | BE | BE | AC | AC | AC | AC | AC | AC | AC | AC | AC | AC | AC |
| Abx- Enema | DFS.001 | BE | BE | ACD |  | ACD | ACD | ACD | ACD | ACD | ACD | ACD | ACD | AC |
|  | DFS.013 | B | BE | ACD |  | ACD | ACD | ACD | ACD | ACD | ACD | ACD | ACD | AC |
|  | DFS.056 | BE | BE | ACD |  | ACD | ACD | ACD | ACD | ACD | ACD | AD | AC |  |
|  | DFS.061 | BE | BE | AC |  |  | AC | AC | AC | AC | AC | AC | AC | AC |
|  | DFS.062 | BE | BE | AC |  | AC | AC | AC | AC | AC | AC | AC | AC | AC |

**Table S2. Antimicrobial resistance determinants (AMRs) and resistance to antibiotics from the antibiotic cocktail.** Each AMR was checked for resistance to vancomycin, neomycin, or metronidazole in <https://www.ncbi.nlm.nih.gov/pathogens/refgene>.

| **AMR** | **Vancomycin** | **Neomycin** | **Metronidazole** |
| --- | --- | --- | --- |
| Aac3-I |  | X |  |
| Aac3-Ib |  | X |  |
| Aac3-IIa |  | X |  |
| AacAad |  | X |  |
| AadA |  |  |  |
| AMPH |  |  |  |
| Aph3Ia |  | X |  |
| CatA1 |  |  |  |
| CatBx |  |  |  |
| CfxA |  |  |  |
| Erm35 |  |  |  |
| ErmB |  |  |  |
| ErmF |  |  |  |
| ErmX |  |  |  |
| MphA |  |  |  |
| MphC |  |  |  |
| MrdA |  |  |  |
| MsrA |  |  |  |
| OqxB |  |  |  |
| StrB |  |  |  |
| TEM-1D |  |  |  |
| Tet-39 |  |  |  |
| Tet-40 |  |  |  |
| TetB |  |  |  |
| TetC |  |  |  |
| TetM |  |  |  |
| TetO |  |  |  |
| TetQ |  |  |  |
| TetW |  |  |  |
| VgaA |  |  |  |

**Table S3. Significant pathways for the genes in the response-associated co-expression modules.** Two, 50, 50, and 20 pathways were significantly enriched for the genes of the salmon (M1), red (M2), dark red (M3), and light green (M4) modules, respectively, at an FDR-adjusted p-value of <0.05.

| **M1: Salmon module (246 genes)** | | | | | |
| --- | --- | --- | --- | --- | --- |
| Name | Source | P-value | B&H adjusted | Genes from Input | Genes in Annotation |
| Post-translational protein modification | BioSystems: REACTOME | 0.00002033 | 0.02722 | 29 | 1037 |
| Fructose metabolism | BioSystems: REACTOME | 0.00006018 | 0.04029 | 3 | 7 |
| **M2: Red module (618 genes)** | | | | | |
| Name | Source | P-value | B&H adjusted | Genes from Input | Genes in Annotation |
| Gene Expression | BioSystems: REACTOME | 7.492E-24 | 1.6E-20 | 140 | 1834 |
| Major pathway of rRNA processing in the nucleolus and cytosol | BioSystems: REACTOME | 4.806E-12 | 5.133E-09 | 28 | 180 |
| rRNA processing | BioSystems: REACTOME | 1.53E-11 | 1.089E-08 | 29 | 202 |
| rRNA processing in the nucleus and cytosol | BioSystems: REACTOME | 2.363E-11 | 1.262E-08 | 28 | 192 |
| Cap-dependent Translation Initiation | BioSystems: REACTOME | 5.473E-10 | 1.948E-07 | 21 | 125 |
| Eukaryotic Translation Initiation | BioSystems: REACTOME | 5.473E-10 | 1.948E-07 | 21 | 125 |
| Formation of a pool of free 40S subunits | BioSystems: REACTOME | 9.989E-10 | 3.048E-07 | 19 | 105 |
| Translation | BioSystems: REACTOME | 2.863E-09 | 7.323E-07 | 23 | 163 |
| Influenza Viral RNA Transcription and Replication | BioSystems: REACTOME | 3.086E-09 | 7.323E-07 | 21 | 137 |
| GTP hydrolysis and joining of the 60S ribosomal subunit | BioSystems: REACTOME | 6.578E-09 | 1.277E-06 | 19 | 117 |
| L13a-mediated translational silencing of Ceruloplasmin expression | BioSystems: REACTOME | 6.578E-09 | 1.277E-06 | 19 | 117 |
| Influenza Life Cycle | BioSystems: REACTOME | 9.951E-09 | 1.771E-06 | 21 | 146 |
| Influenza Infection | BioSystems: REACTOME | 3.674E-08 | 5.831E-06 | 21 | 157 |
| Peptide chain elongation | BioSystems: REACTOME | 3.822E-08 | 5.831E-06 | 16 | 92 |
| Selenocysteine synthesis | BioSystems: REACTOME | 6.117E-08 | 8.711E-06 | 16 | 95 |
| Eukaryotic Translation Termination | BioSystems: REACTOME | 7.125E-08 | 9.512E-06 | 16 | 96 |
| Selenoamino acid metabolism | BioSystems: REACTOME | 7.645E-08 | 9.605E-06 | 18 | 122 |
| Eukaryotic Translation Elongation | BioSystems: REACTOME | 8.282E-08 | 9.828E-06 | 16 | 97 |
| Activation of the mRNA upon binding of the cap-binding complex and eIFs, and subsequent binding to 43S | BioSystems: REACTOME | 8.944E-08 | 0.00001006 | 13 | 63 |
| Formation of the ternary complex, and subsequently, the 43S complex | BioSystems: REACTOME | 9.457E-08 | 0.0000101 | 12 | 53 |
| Nonsense Mediated Decay (NMD) independent of the Exon Junction Complex (EJC) | BioSystems: REACTOME | 1.112E-07 | 0.00001131 | 16 | 99 |
| Ribosome | MSigDB C2 BIOCARTA (v7.5.1) | 1.346E-07 | 0.00001307 | 15 | 88 |
| Processing of Capped Intron-Containing Pre-mRNA | BioSystems: REACTOME | 1.424E-07 | 0.00001323 | 26 | 248 |
| Cytoplasmic ribosomal proteins | MSigDB C2 BIOCARTA (v7.5.1) | 1.83E-07 | 0.00001629 | 15 | 90 |
| Viral mRNA Translation | BioSystems: REACTOME | 2.467E-07 | 0.00002108 | 15 | 92 |
| Nonsense-Mediated Decay (NMD) | BioSystems: REACTOME | 3.208E-07 | 0.00002538 | 17 | 120 |
| Nonsense Mediated Decay (NMD) enhanced by the Exon Junction Complex (EJC) | BioSystems: REACTOME | 3.208E-07 | 0.00002538 | 17 | 120 |
| Ribosomal scanning and start codon recognition | BioSystems: REACTOME | 4.879E-07 | 0.00003722 | 12 | 61 |
| Ribosome | BioSystems: KEGG | 5.352E-07 | 0.00003942 | 19 | 153 |
| Translation initiation complex formation | BioSystems: REACTOME | 5.874E-07 | 0.00004183 | 12 | 62 |
| Infectious disease | BioSystems: REACTOME | 1.301E-06 | 0.00008964 | 32 | 388 |
| SRP-dependent cotranslational protein targeting to membrane | BioSystems: REACTOME | 4.558E-06 | 0.0003042 | 15 | 115 |
| Aminoacyl-tRNA biosynthesis | MSigDB C2 BIOCARTA (v7.5.1) | 5.151E-06 | 0.0003334 | 9 | 41 |
| mRNA Splicing | BioSystems: REACTOME | 0.00000602 | 0.0003782 | 20 | 196 |
| Rev-mediated nuclear export of HIV RNA | BioSystems: REACTOME | 0.00001003 | 0.0006123 | 8 | 34 |
| tRNA processing | BioSystems: REACTOME | 0.00001133 | 0.0006703 | 14 | 109 |
| tRNA Aminoacylation | BioSystems: REACTOME | 0.00001161 | 0.0006703 | 9 | 45 |
| Cell Cycle, Mitotic | BioSystems: REACTOME | 0.00001273 | 0.0007156 | 36 | 515 |
| Metabolism of proteins | BioSystems: REACTOME | 0.00001507 | 0.0008254 | 83 | 1624 |
| Interactions of Rev with host cellular proteins | BioSystems: REACTOME | 0.00001579 | 0.0008434 | 8 | 36 |
| rRNA modification in the nucleus and cytosol | BioSystems: REACTOME | 0.00003225 | 0.00168 | 10 | 63 |
| DNA Repair | BioSystems: REACTOME | 0.00004039 | 0.002054 | 25 | 317 |
| mRNA Splicing - Minor Pathway | BioSystems: REACTOME | 0.00004613 | 0.002291 | 9 | 53 |
| Metabolism of amino acids and derivatives | BioSystems: REACTOME | 0.00005539 | 0.002689 | 27 | 363 |
| Cell Cycle | BioSystems: REACTOME | 0.00006395 | 0.002988 | 39 | 622 |
| Spliceosome | MSigDB C2 BIOCARTA (v7.5.1) | 0.00006435 | 0.002988 | 14 | 127 |
| Mitochondrial tRNA aminoacylation | BioSystems: REACTOME | 0.00007155 | 0.003251 | 6 | 23 |
| M Phase | BioSystems: REACTOME | 0.0000731 | 0.003253 | 24 | 309 |
| Spliceosome | BioSystems: KEGG | 0.0001155 | 0.005036 | 14 | 134 |
| mRNA Splicing - Major Pathway | BioSystems: REACTOME | 0.0001363 | 0.005822 | 17 | 188 |
| **M3: Dark red module (69 genes)** | | | | | |
| Name | Source | P-value | B&H adjusted | Genes from Input | Genes in Annotation |
| Metabolic pathways | BioSystems: KEGG | 0.00005386 | 0.01244 | 15 | 1272 |
| Metabolism of amino acids and derivatives | BioSystems: REACTOME | 0.000064 | 0.01244 | 8 | 363 |
| SALM protein interactions at the synapses | BioSystems: REACTOME | 0.00006666 | 0.01244 | 3 | 21 |
| Branched-chain amino acid catabolism | BioSystems: REACTOME | 0.0001006 | 0.01409 | 3 | 24 |
| glycolysis/gluconeogenesis | Pathway Ontology | 0.0001792 | 0.02007 | 3 | 29 |
| MAP00680 Methane metabolism | GenMAPP | 0.0002971 | 0.02773 | 2 | 7 |
| Mitochondrial translation elongation | BioSystems: REACTOME | 0.0003641 | 0.02913 | 4 | 89 |
| Mitochondrial translation | BioSystems: REACTOME | 0.0004668 | 0.03268 | 4 | 95 |
| leucine degradation | BioSystems: BIOCYC | 0.0009223 | 0.04475 | 2 | 12 |
| Carbon metabolism | BioSystems: KEGG | 0.0009276 | 0.04475 | 4 | 114 |
| Amyotrophic lateral sclerosis (ALS) | BioSystems: KEGG | 0.0009628 | 0.04475 | 3 | 51 |
| Amyotrophic lateral sclerosis (ALS) | MSigDB C2 BIOCARTA (v7.5.1) | 0.001077 | 0.04475 | 3 | 53 |
| Leucine degradation, leucine => acetoacetate + acetyl-CoA | BioSystems: KEGG | 0.001087 | 0.04475 | 2 | 13 |
| Transcriptional activation by NRF2 in response to phytochemicals | MSigDB C2 BIOCARTA (v7.5.1) | 0.001457 | 0.04475 | 2 | 15 |
| Glycolysis / Gluconeogenesis | MSigDB C2 BIOCARTA (v7.5.1) | 0.001698 | 0.04475 | 3 | 62 |
| CREB phosphorylation through the activation of CaMKII | BioSystems: REACTOME | 0.001877 | 0.04475 | 2 | 17 |
| Unblocking of NMDA receptor, glutamate binding and activation | BioSystems: REACTOME | 0.001877 | 0.04475 | 2 | 17 |
| Tryptophan Metabolism | SMPDB | 0.001877 | 0.04475 | 2 | 17 |
| Glycolysis / Gluconeogenesis | BioSystems: KEGG | 0.002122 | 0.04475 | 3 | 67 |
| Ras activation uopn Ca2+ infux through NMDA receptor | BioSystems: REACTOME | 0.002349 | 0.04475 | 2 | 19 |
| SOS-mediated signalling | BioSystems: REACTOME | 0.002368 | 0.04475 | 5 | 245 |
| RAF/MAP kinase cascade | BioSystems: REACTOME | 0.002368 | 0.04475 | 5 | 245 |
| GRB2 events in EGFR signaling | BioSystems: REACTOME | 0.002368 | 0.04475 | 5 | 245 |
| SHC1 events in EGFR signaling | BioSystems: REACTOME | 0.002368 | 0.04475 | 5 | 245 |
| ARMS-mediated activation | BioSystems: REACTOME | 0.00254 | 0.04475 | 5 | 249 |
| Signalling to p38 via RIT and RIN | BioSystems: REACTOME | 0.00254 | 0.04475 | 5 | 249 |
| Frs2-mediated activation | BioSystems: REACTOME | 0.002585 | 0.04475 | 5 | 250 |
| Protein-protein interactions at synapses | BioSystems: REACTOME | 0.002606 | 0.04475 | 3 | 72 |
| MAPK1/MAPK3 signaling | BioSystems: REACTOME | 0.002629 | 0.04475 | 5 | 251 |
| Prolonged ERK activation events | BioSystems: REACTOME | 0.002675 | 0.04475 | 5 | 252 |
| Signaling by Leptin | BioSystems: REACTOME | 0.002721 | 0.04475 | 5 | 253 |
| Interleukin receptor SHC signaling | BioSystems: REACTOME | 0.002815 | 0.04475 | 5 | 255 |
| Signalling to RAS | BioSystems: REACTOME | 0.002863 | 0.04475 | 5 | 256 |
| Nitric Oxide Signaling Pathway | MSigDB C2 BIOCARTA (v7.5.1) | 0.002871 | 0.04475 | 2 | 21 |
| VEGFR2 mediated cell proliferation | BioSystems: REACTOME | 0.002961 | 0.04475 | 5 | 258 |
| Interleukin-2 signaling | BioSystems: REACTOME | 0.003163 | 0.04475 | 5 | 262 |
| Signalling to ERKs | BioSystems: REACTOME | 0.003215 | 0.04475 | 5 | 263 |
| tryptophan metabolic | Pathway Ontology | 0.003442 | 0.04475 | 2 | 23 |
| NRF2-ARE regulation | MSigDB C2 BIOCARTA (v7.5.1) | 0.003442 | 0.04475 | 2 | 23 |
| RET signaling | BioSystems: REACTOME | 0.003597 | 0.04475 | 5 | 270 |
| Interleukin-3, 5 and GM-CSF signaling | BioSystems: REACTOME | 0.003654 | 0.04475 | 5 | 271 |
| FCERI mediated MAPK activation | BioSystems: REACTOME | 0.003654 | 0.04475 | 5 | 271 |
| Metabotropic glutamate receptor group I pathway | PantherDB | 0.003745 | 0.04475 | 2 | 24 |
| FGFR4 mutant receptor activation | BioSystems: REACTOME | 0.003835 | 0.04475 | 1 | 1 |
| methane metabolic | Pathway Ontology | 0.003835 | 0.04475 | 1 | 1 |
| oxidative stress responses | Pathway Ontology | 0.003835 | 0.04475 | 1 | 1 |
| Isovaleric Aciduria | SMPDB | 0.003835 | 0.04475 | 1 | 1 |
| Carbamoyl Phosphate Synthetase Deficiency | SMPDB | 0.003835 | 0.04475 | 1 | 1 |
| NCAM signaling for neurite out-growth | BioSystems: REACTOME | 0.00395 | 0.04514 | 5 | 276 |
| MAP00280 Valine leucine and isoleucine degradation | GenMAPP | 0.004389 | 0.04854 | 2 | 26 |
| **M4: Light green module (121 genes)** | | | | | |
| Name | Source | P-value | B&H adjusted | Genes from Input | Genes in Annotation |
| Cytokine Signaling in Immune system | BioSystems: REACTOME | 3.727E-06 | 0.003492 | 18 | 760 |
| Hemostasis | BioSystems: REACTOME | 6.964E-06 | 0.003492 | 16 | 639 |
| Signaling events mediated by VEGFR1 and VEGFR2 | MSigDB C2 BIOCARTA (v7.5.1) | 7.806E-06 | 0.003492 | 6 | 69 |
| Platelet activation, signaling and aggregation | BioSystems: REACTOME | 0.00002428 | 0.008144 | 10 | 282 |
| Signaling events mediated by VEGFR1 and VEGFR2 | BioSystems: Pathway Interaction Database | 0.00007235 | 0.01676 | 5 | 63 |
| Alpha4 beta1 integrin signaling events | MSigDB C2 BIOCARTA (v7.5.1) | 0.00007492 | 0.01676 | 4 | 33 |
| Rap1 signaling pathway | BioSystems: KEGG | 0.0001005 | 0.01926 | 8 | 210 |
| Gastrin signaling pathway | MSigDB C2 BIOCARTA (v7.5.1) | 0.0001354 | 0.02271 | 6 | 114 |
| Influenza A | BioSystems: KEGG | 0.000192 | 0.02863 | 7 | 173 |
| Signaling by Interleukins | BioSystems: REACTOME | 0.0002702 | 0.03398 | 12 | 528 |
| Platelet degranulation | BioSystems: REACTOME | 0.000301 | 0.03398 | 6 | 132 |
| Salmonella infection | BioSystems: KEGG | 0.0003164 | 0.03398 | 5 | 86 |
| Translation inhibitors in chronically activated PDGFRA cells | MSigDB C2 BIOCARTA (v7.5.1) | 0.0003291 | 0.03398 | 4 | 48 |
| Response to elevated platelet cytosolic Ca2+ | BioSystems: REACTOME | 0.0003675 | 0.03523 | 6 | 137 |
| Role of Calcineurin-dependent NFAT signaling in lymphocytes | BioSystems: Pathway Interaction Database | 0.0004161 | 0.03632 | 4 | 51 |
| SARS-CoV-2 altering angiogenesis via NRP1 | MSigDB C2 BIOCARTA (v7.5.1) | 0.0004657 | 0.03632 | 2 | 5 |
| Role of Calcineurin-dependent NFAT signaling in lymphocytes | MSigDB C2 BIOCARTA (v7.5.1) | 0.0004825 | 0.03632 | 4 | 53 |
| Innate Immune System | BioSystems: REACTOME | 0.0004871 | 0.03632 | 20 | 1302 |
| VEGF signaling pathway | PantherDB | 0.0006809 | 0.04656 | 4 | 58 |
| Apoptosis signaling pathway | PantherDB | 0.0006939 | 0.04656 | 5 | 102 |

**Table S4. Comparisons of alpha diversity between non-responders, responders, and donors.** Comparisons were performed using a Wilcoxon rank sum test and significant differences are in bold. NR, non-responder; R, responder; D, donor; ABX, antibiotic; FMT, fecal microbial transplantation; F/u, follow-up.

|  | **N** | **NR vs R** | **NR vs D** | **R vs D** |
| --- | --- | --- | --- | --- |
| Baseline | 18 | 0.14 | **0.03** | 0.47 |
| Post-ABX | 9 | 0.10 | 0.10 | **0.02** |
| Post-FMT1 | 15 | 0.46 | **0.02** | 0.13 |
| Post-FMT2 | 17 | **0.04** | 0.08 | 0.86 |
| Post-FMT3 | 19 | 0.07 | 0.06 | 0.93 |
| Post-FMT4 | 18 | 0.08 | **0.02** | 0.60 |
| Post-FMT5 | 19 | 0.09 | 0.06 | 0.37 |
| Post-FMT6 | 15 | 0.40 | 0.18 | 0.38 |
| F/u-1 | 18 | **0.03** | 0.10 | 1.0 |
| F/u-2 | 18 | **5.6x10^-3^** | **9.0x10^-3^** | 0.86 |
| F/u-3 | 13 | 0.13 | 0.25 | 0.63 |

**Table S5. Microbiome-derived metabolites associated with microbial species increased in responders.** Integration of fecal shotgun metagenomic (*HumaNn3*) and metabolomic datasets (Metabolon) was performed using *MIMOSA2*. All level4ec functions were converted into KEGG functions and those functions found in *Bacteroides dorei*, *Blautia hydrogenotrophica*, and *Blautia hydrogenotrophica* *CAG 147* were used as input for the metagenomic dataset. Normalized abundance levels for 481 metabolites with KEGG IDs were used as input for the metabolomic dataset. The FDR-adjusted p-value is shown for the correlation between the community metabolic potential score (based on the stratified functions in the metagenomic data) and the normalized metabolite abundance levels. The species that encode genes that either produce or metabolize (utilize) the metabolite are listed. The common name for the gene is also listed based on KEGG ORTHOLOGY.

| **Metabolite** | **FDR-adjusted p-value** | **Producing taxa; genes/reactions** | **Utilizing taxa; gene/reaction** |
| --- | --- | --- | --- |
| L-Arginine | 0.046 |  | *Bacteroides dorei*; arginine decarboxylase (K01583); arginase (K01476) |
|  |  |  | *Blautia hydrogenotrophica*; arginine decarboxylase (K01583); arginase (K01476) |
|  |  |  | *Blautia hydrogenotrophica CAG 147*; arginine decarboxylase (K01583); arginase (K01476) |
|  |  |  | *Butyricimonas virosa*; arginine decarboxylase (K01583); arginase (K01476) |
| 4-Coumarate | 0.037 |  | *Blautia hydrogenotrophica*; flavin prenyltransferase (K03176) |
|  |  |  | *Blautia hydrogenotrophica CAG 147*; flavin prenyltransferase (K03176) |

**Table S6. Microbiome-derived metabolites associated with microbial species decreased in responders.** Integration of fecal shotgun metagenomic (*HumaNn3*) and metabolomic datasets (Metabolon) was performed using *MIMOSA2*. All level4ec functions were converted into KEGG functions and those functions found in *Bacteroides fragilis*, *Bacteroides fragilis CAG 47*, and *Bacteroides salyersiae* were used as input for the metagenomic dataset. Normalized abundance levels for 481 metabolites with KEGG IDs were used as input for the metabolomic dataset. The FDR-adjusted p-value is shown for the correlation between the community metabolic potential score (based on the stratified functions in the metagenomic data) and the normalized metabolite abundance levels. The species that encode genes that either produce or metabolize (utilize) the metabolite are listed. The common name for the gene is also listed based on KEGG ORTHOLOGY.

| **Metabolite** | **FDR-adjusted P-value** | **Producing taxa; genes/reactions** | **Utilizing taxa; gene/reaction** |
| --- | --- | --- | --- |
| Taurine | 0.036 | *Bacteroides fragilis*; glutamate decarboxylase (K01580), choloylglycine (K01442) |  |
|  |  | *Bacteroides fragilis CAG 47*; glutamate decarboxylase (K01580), choloylglycine (K01442) |  |
|  |  | *Bacteroides salyersiae*; glutamate decarboxylase (K01580) |  |
| Alanine | 0.041 | *Bacteroides fragilis*; aspartate 4-decarboxylase (K09758) |  |
|  |  | *Bacteroides fragilis CAG 47*; aspartate 4-decarboxylase (K09758) |  |
| Choline | 0.05 | *Bacteroides fragilis*; glycerophosphoryl diester phosphodiesterase (K01126) |  |
|  |  | *Bacteroides fragilis CAG 47*; glycerophosphoryl diester phosphodiesterase(K01126) |  |
| Hypotaurine | 0.05 | *Bacteroides fragilis*; glutamate decarboxylase (K01580) |  |
|  |  | *Bacteroides fragilis CAG 47*; glutamate decarboxylase (K01580) |  |
|  |  | *Bacteroides salyersiae*; glutamate decarboxylase (K01580) |  |

**
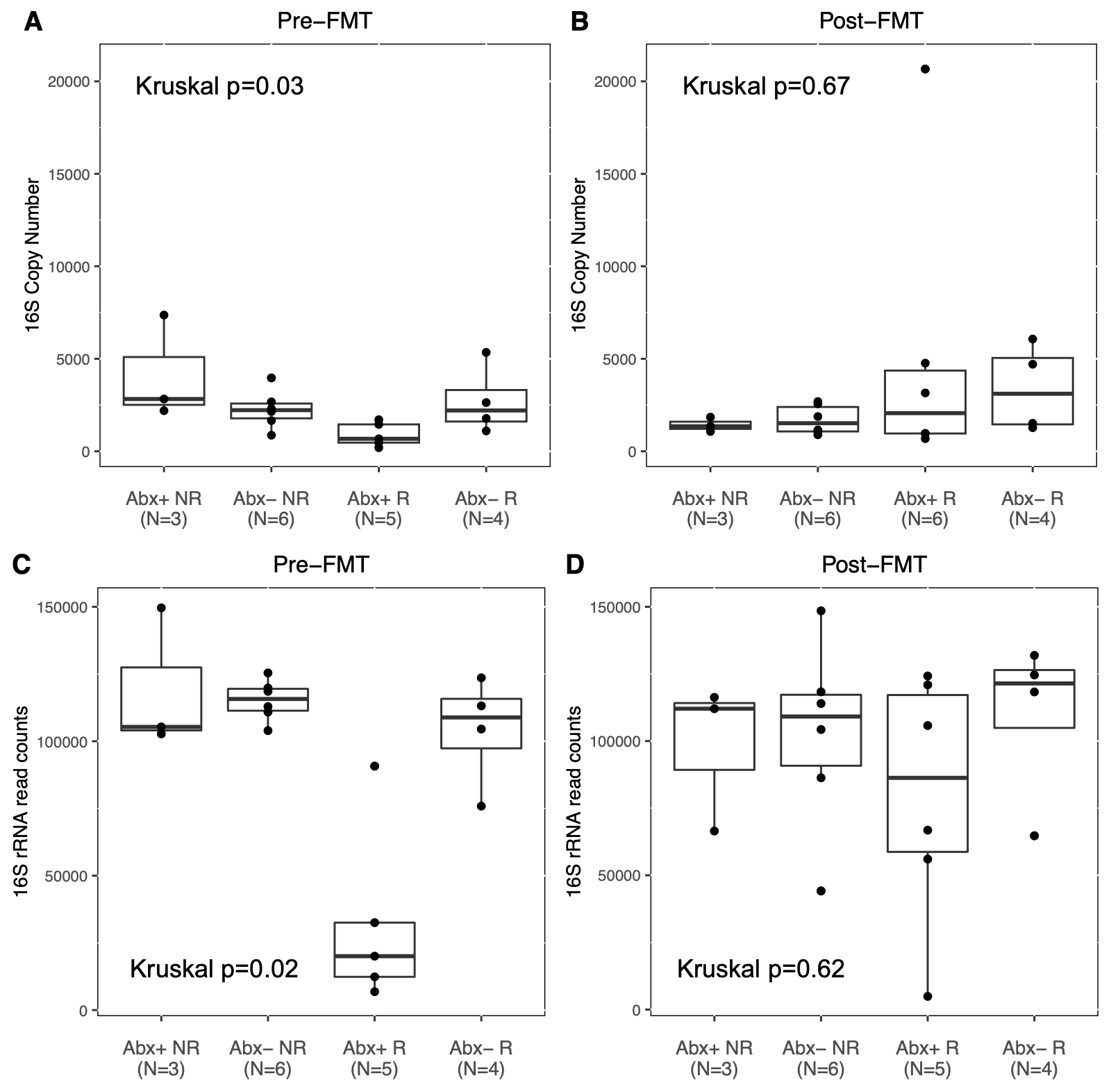
**

**Figure S1. 16S rRNA copy number and read count of pre- and post-FMT colonic mucosal-associated microbiota.**

16S rRNA copy number was compared between antibiotic response groups **(A)** before FMT (N=18) and **(B)** after FMT (N=19). 16S rRNA read count was compared between antibiotic response groups **(C)** before FMT (N=18) and **(D)** after FMT

(N=19). RNA extracts were collected from 19 patients before and after FMT; however, one pre-FMT extract from one patient was of poor quality and not subjected to 16S rRNA copy number or 16S rRNA sequencing. Comparisons between groups

were made using a Kruskal-Wallis test. FMT, fecal microbial transplantation; Abx, antibiotic; NR, non-responder; R, responder.

**
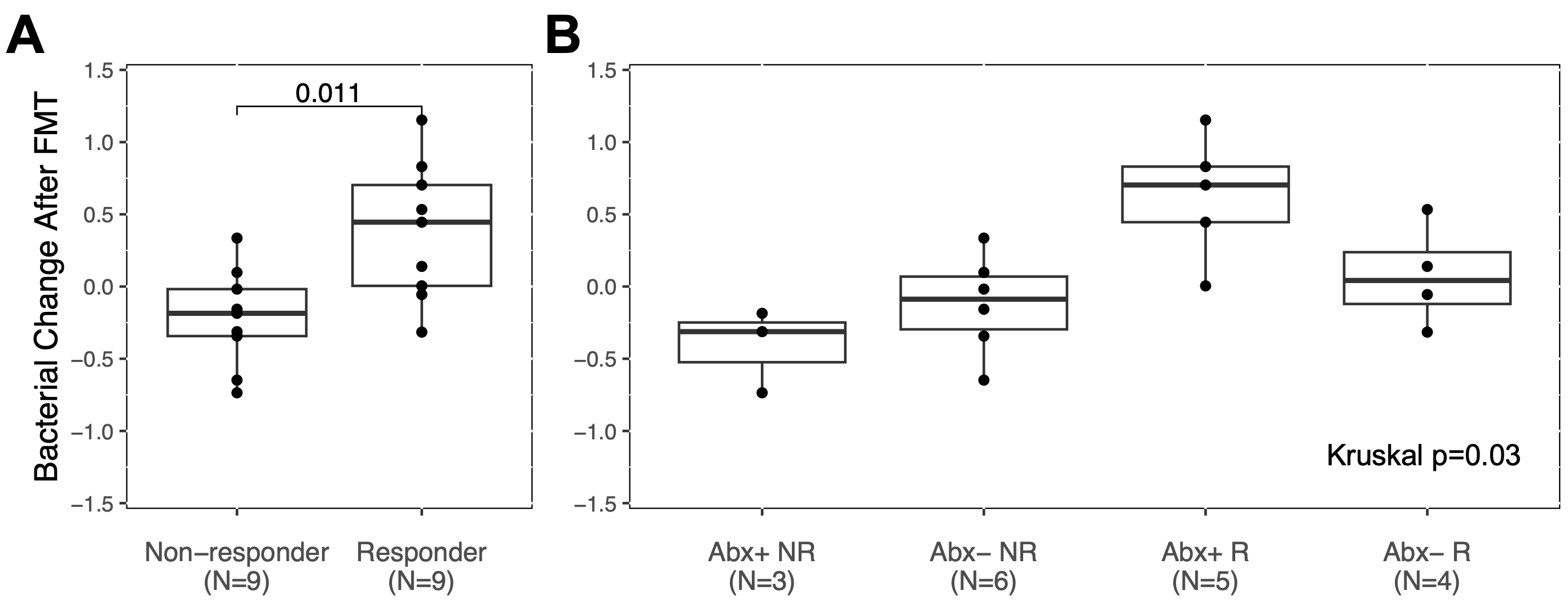
**

**Figure S2. Difference in 16S rRNA copy number from pre- and post-FMT mucosal samples by response and antibiotic pretreatment.** Bacterial change after FMT is the difference in log-transformed 16S rRNA copy number between pre- and post-FMT samples. Comparisons were performed (**A**) using a Wilcoxon rank sum test between non-responders

and responders or (**B**) using a Kruskal-Wallis test between four antibiotic response groups. Abx, antibiotic; NR, non-responder; R, responder.

**
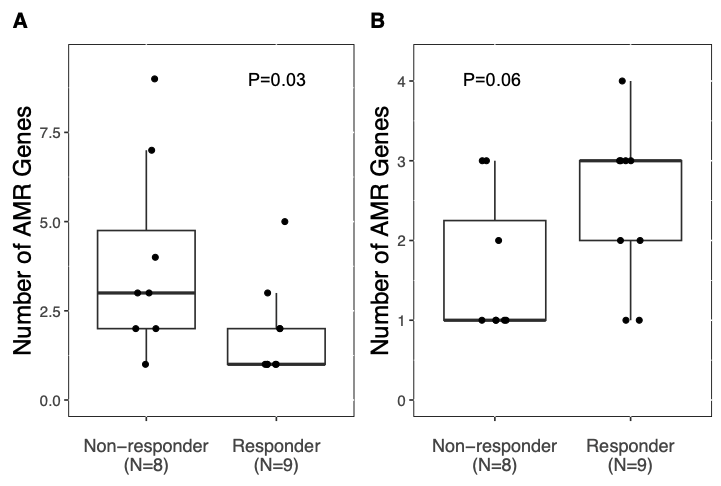
**

**Figure S3. Number of antimicrobial resistance genes (AMR) in pre- and post-FMT colonic mucosal-associated microbiomes by response.** Comparisons between non-responders and responders were performed using a one-sided Wilcoxon rank sum test **(A)** before and **(B)** after FMT.

**
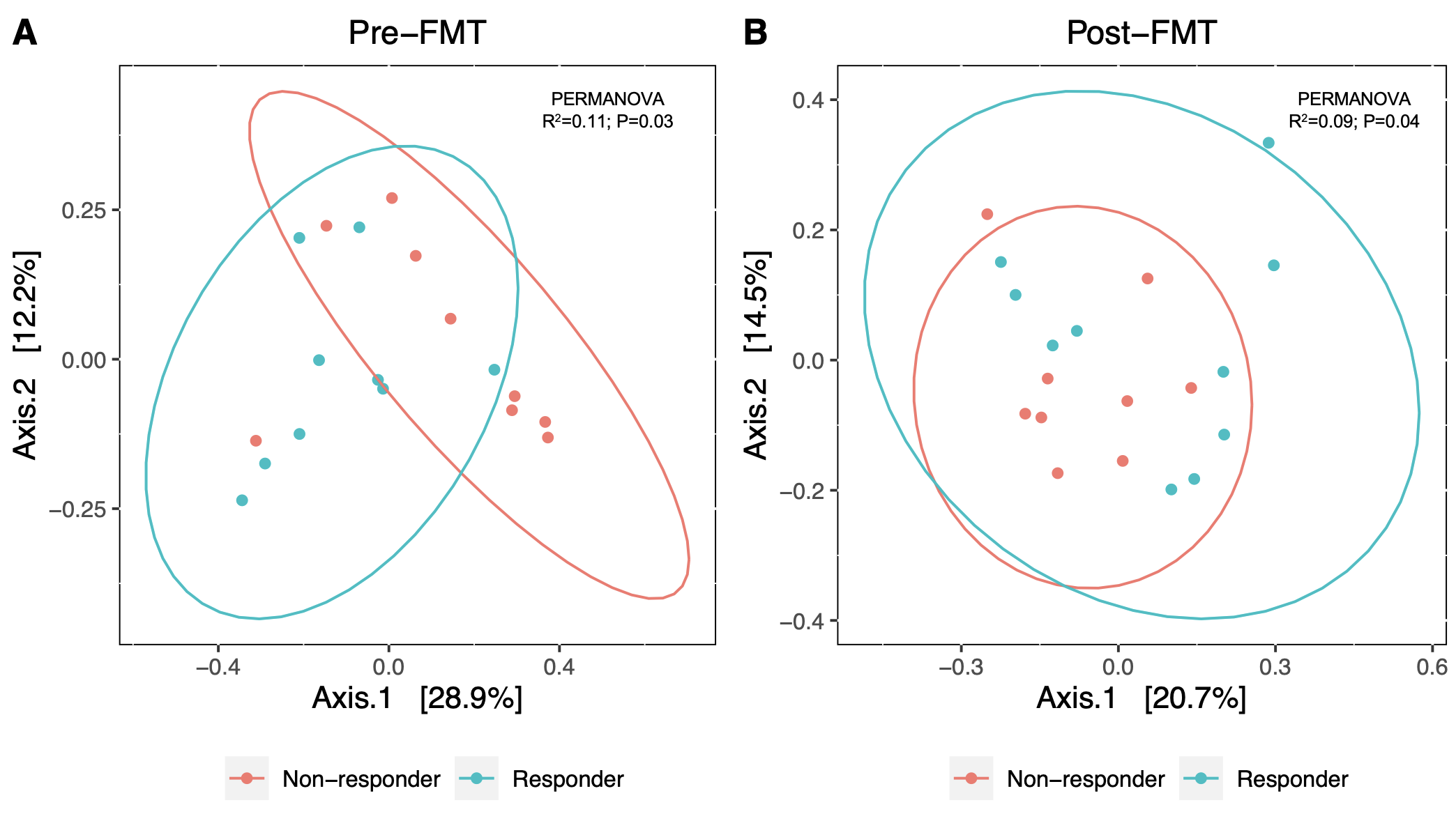
**

**Figure S4. Beta diversity differs between non-responders and responders.** Principal Coordinates Analysis plot of colonic mucosal samples colored by response group. Beta diversity (Unweighted Unifrac) was compared between non-responders and responders using PERMANOVA (**A**) before and (**B**) after FMT.


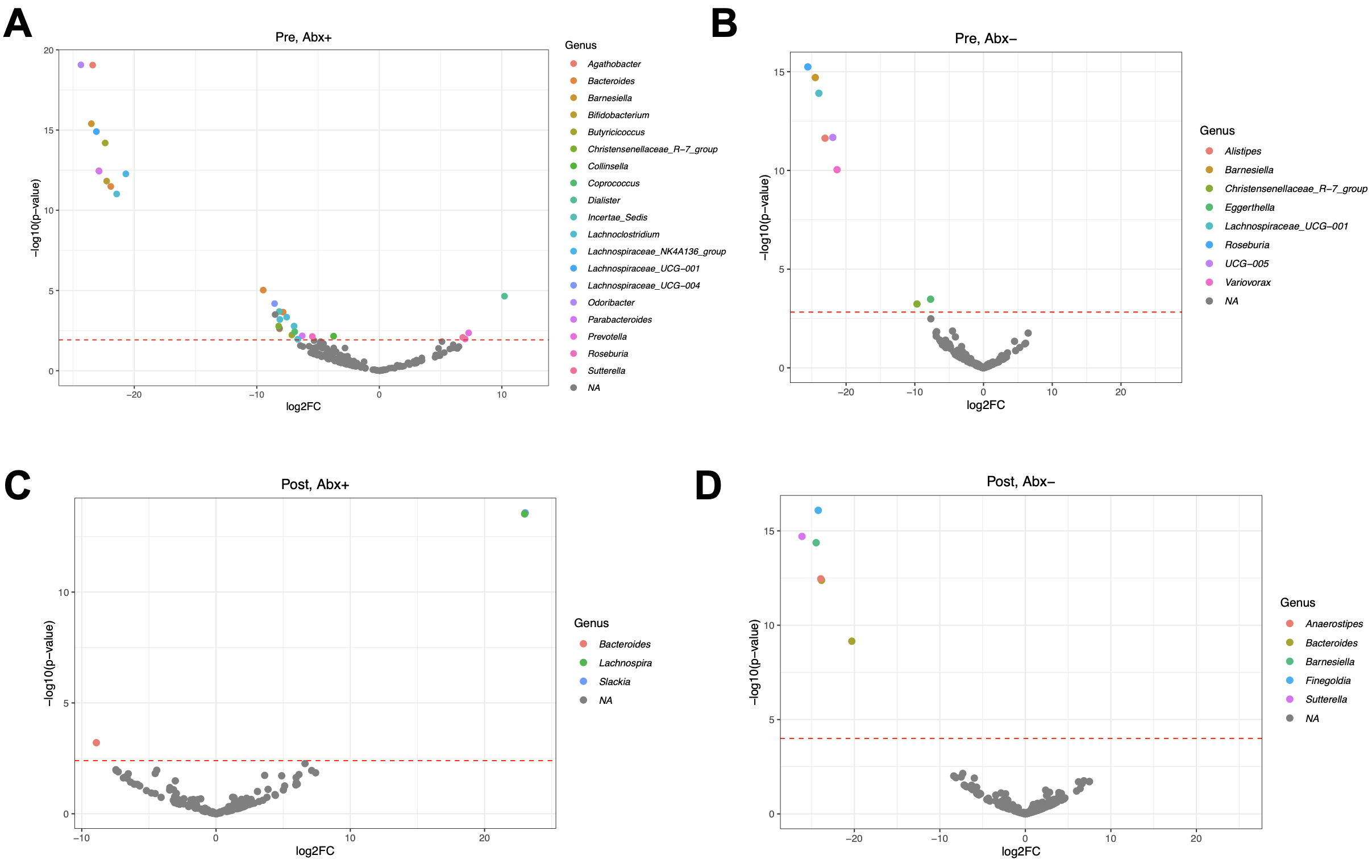
s

*Lachnospira*

*Slackia*

*Agathobacter*

*Odoribacter*

*Barnesiella*

*Roseburia*

*Barnesiella*

*Lachnospiraceae_UCG-001*

*Finegoldia*

*Sutterella*

*Barnesiella*

**Figure S5. Antibiotic pre-treated FMT-responsive patients have the greatest number of differentially abundant taxa.** Four different subsets of FMT-responsive patients (stratified by time point and antibiotic pretreatment) were compared to all non-responders (N=9). Volcano plots are displayed for the **(A)** antibiotic pretreated responders pre-FMT (Pre, Abx+; N=5), **(B)** non-antibiotic pretreated responders Pre-FMT (Pre, Abx-; N=4), **(C)** antibiotic pretreated responders post-FMT (Post, Abx+; N=6), and **(D)** non-antibiotic pretreated responders post-FMT (Post, Abx-; N=4) that were compared to all non-responders at respective time points. Colors indicate genera and “NA” indicates ASVs that were not differentially abundant. ASVs with log2 fold change (FC) > 0 are enriched in responders.


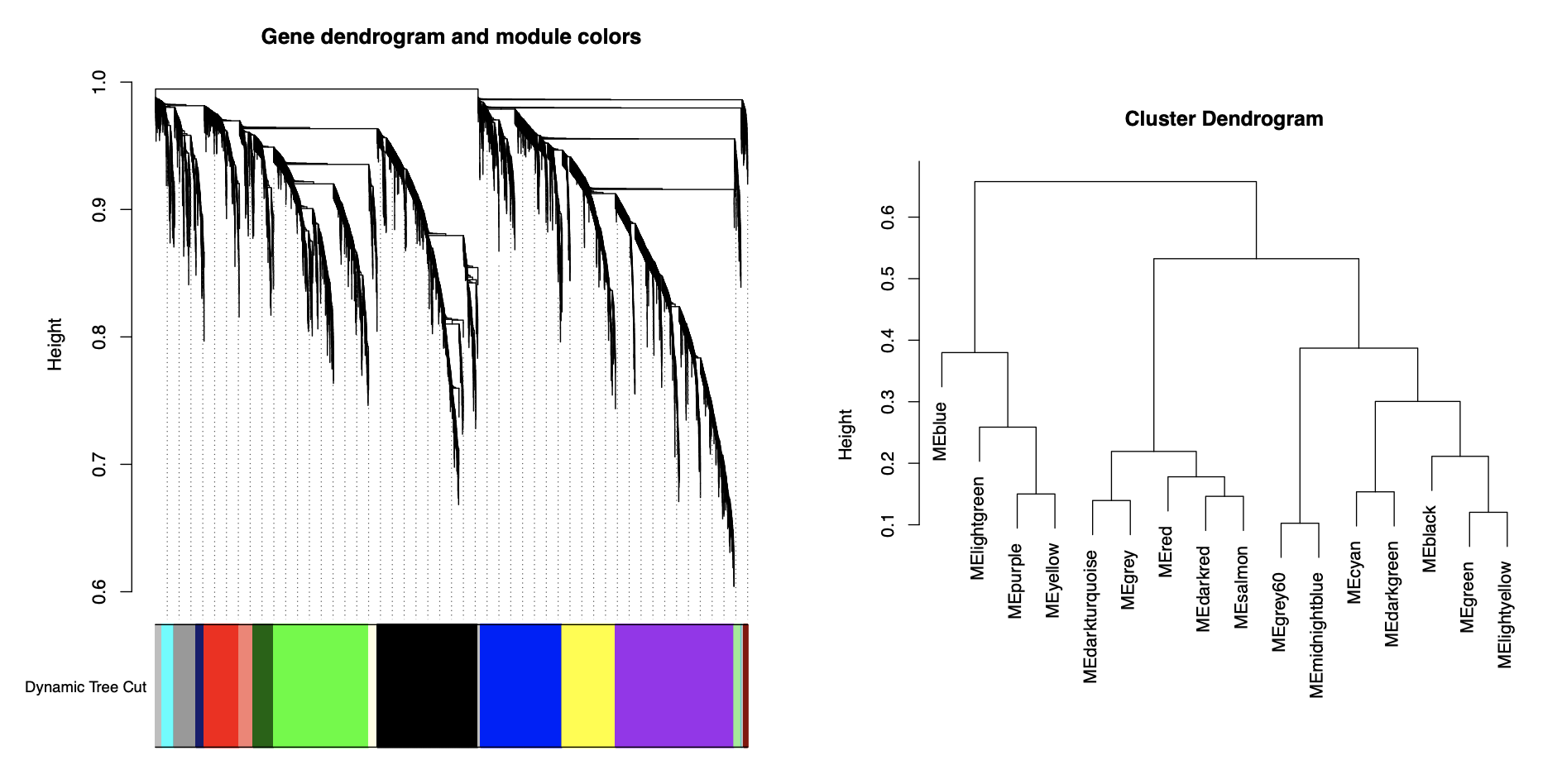


**Figure S6. Cluster dendrogram of all genes and co-expression modules of the intestinal mucosa.** Of 10,473 genes, 10,284 (98.4%) formed 15 WGCNA modules; 189 genes were uncorrelated (gray module).


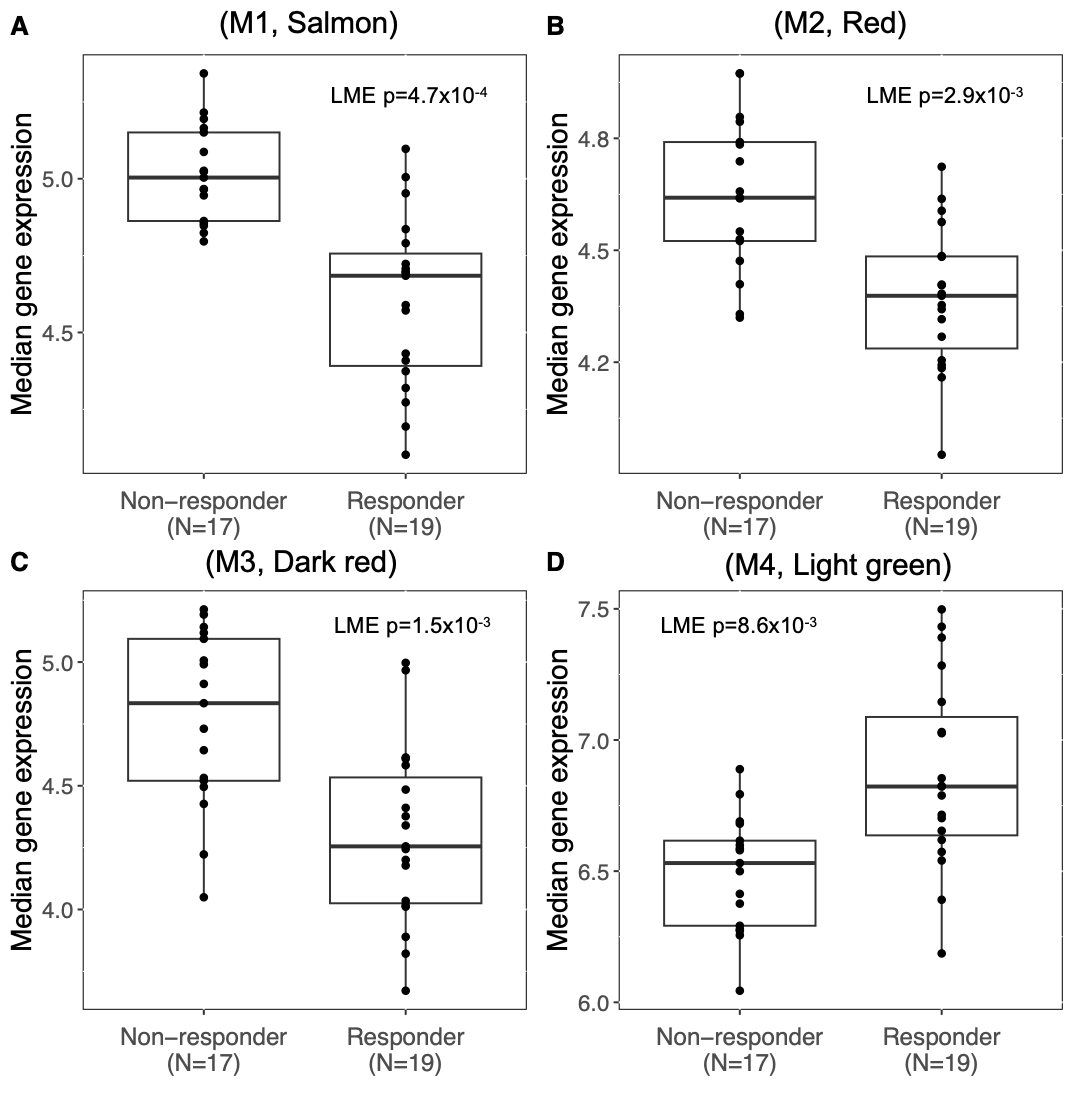


**Figure S7. Median expression of genes in the response-associated co-expression modules after adjusting for multiple testing (15 tests).** The (**A**) salmon, (**B**) red, (**C**) dark red, and (**D**) light green modules were assigned the labels, M1-M4, and contain 246, 618, 69, and 121 co-expressed genes. Associations between response and median gene expression were performed using linear mixed models, adjusting for patient, and p-values are reported in each boxplot.


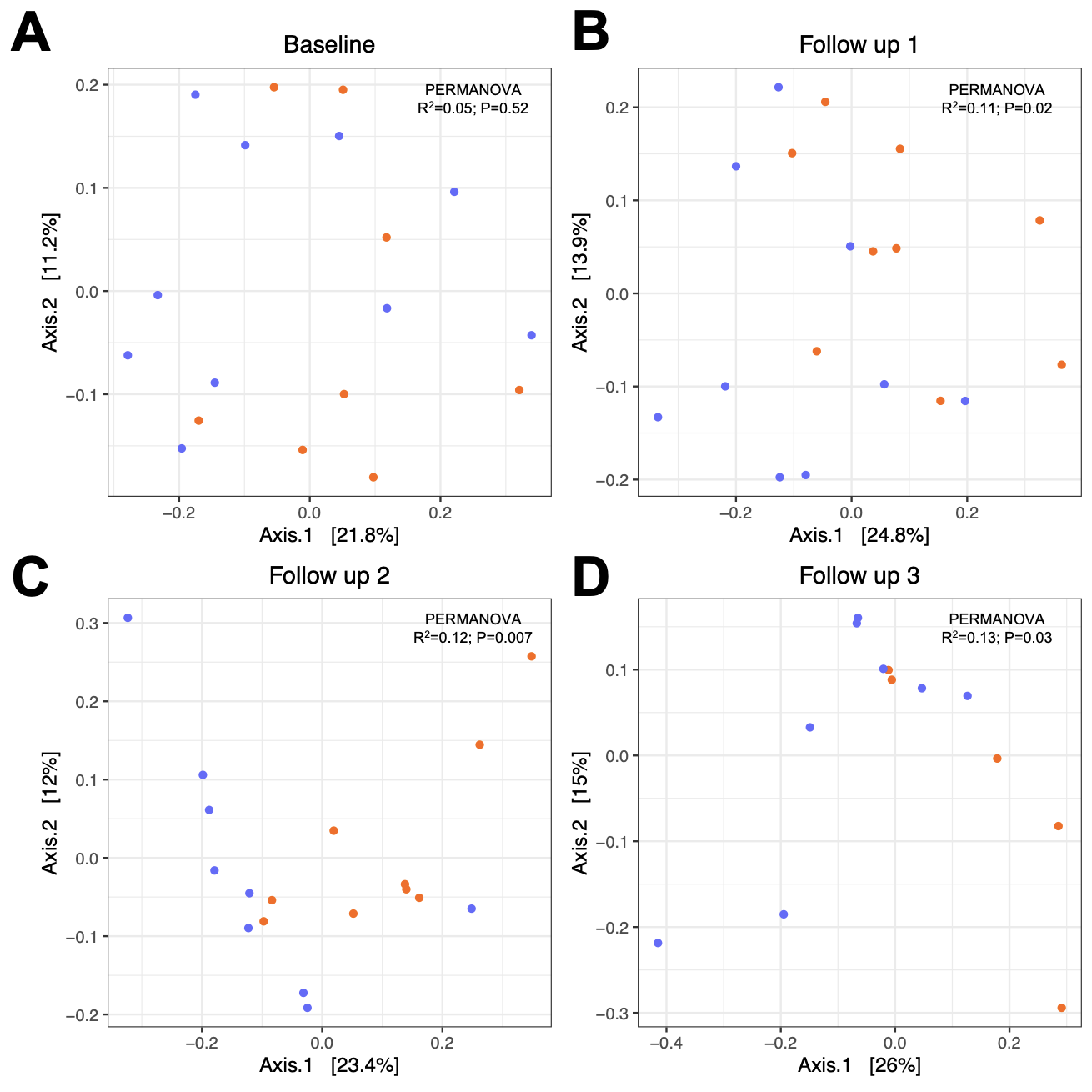


**Figure S8. Fecal microbiota compositional differences between non-responders and responders at all follow-up time points.** Beta diversity (Unweighted UniFrac) was compared between non-responders and responders at **(A)** baseline, **(B)** the first follow-

up time point, **(C)** the second follow-up time point, and **(D)** the third follow-up time point using PERMANOVA.


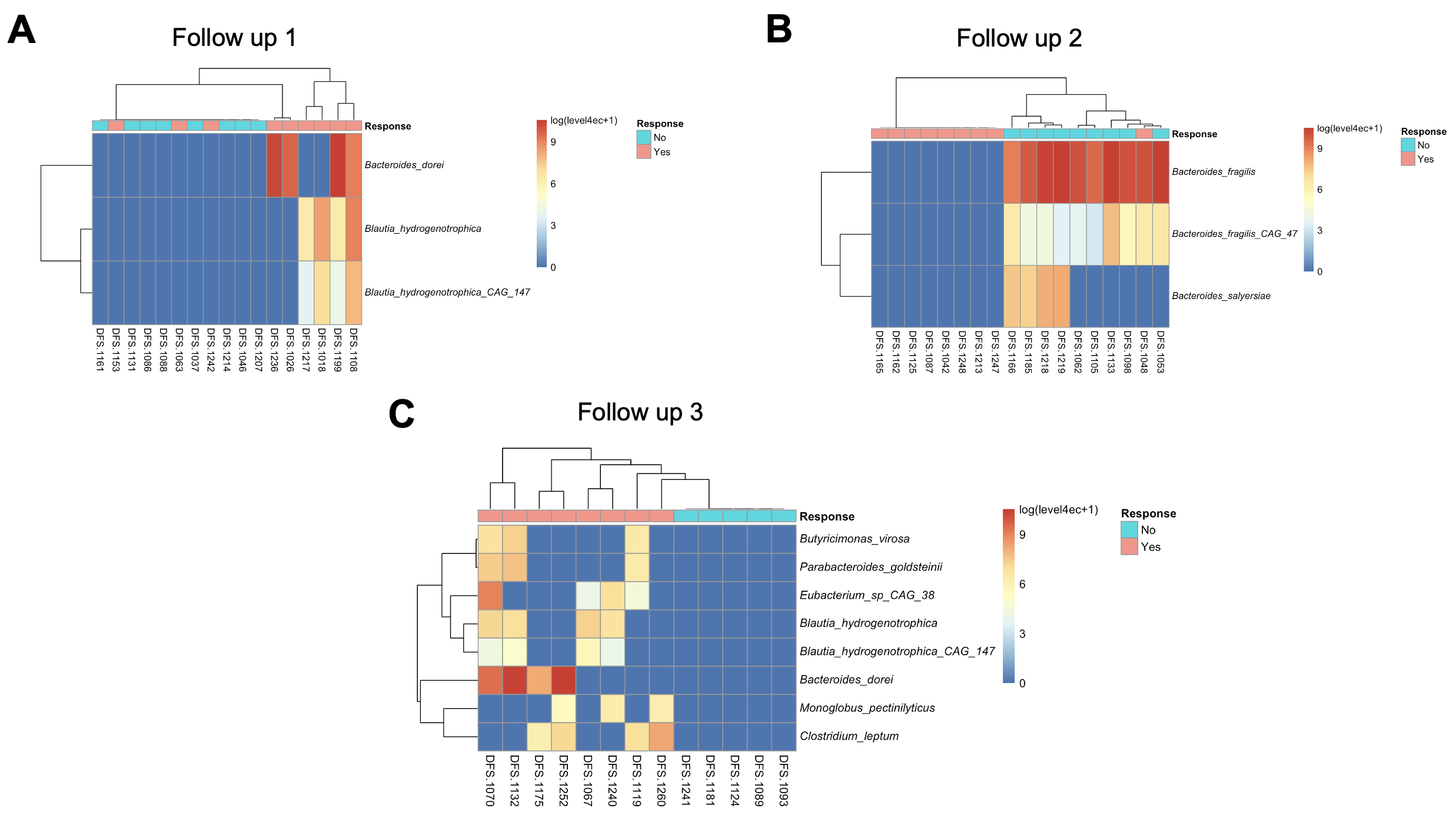


**Figure S9. Fecal microbial species that relate to FMT responsiveness following cessation of the clinical trial.** Heatmaps show the response-associated fecal microbial species and their relative abundances by response group at **(A)** the first follow-up time point, **(B)** the second follow-up time point, and **(C)** the third follow-up time point (FDR-adjusted

p<0.05). Two approaches were performed to identify fecal microbial species that associated with response (see **Methods**): differential abundance analyses and correlation analysis with PC1. Differential abundance of microbial species revealed

three, one, and eight differentially abundance species at the first, second, and third follow-up time points, respectively (**Fig. 3D**). *Bacteroides dorei*, *Blautia hydrogenotrophica*, and *Blautia hydrogenotrophica CAG 147* were present only in

responders compared to non-responders at both first and third follow-up time points. Only *Bacteroides salyersiae* was differentially abundant at follow up 2 and was present only in non-responders compared to responders. Two additional

species, *Bacteroides fragilis* and *Bacteroides fragilis CAG 47*, were correlated with PC1 at the second follow-up time point (Spearman; FDR-adjusted p<0.05), which were decreased in responders compared to non-responders.


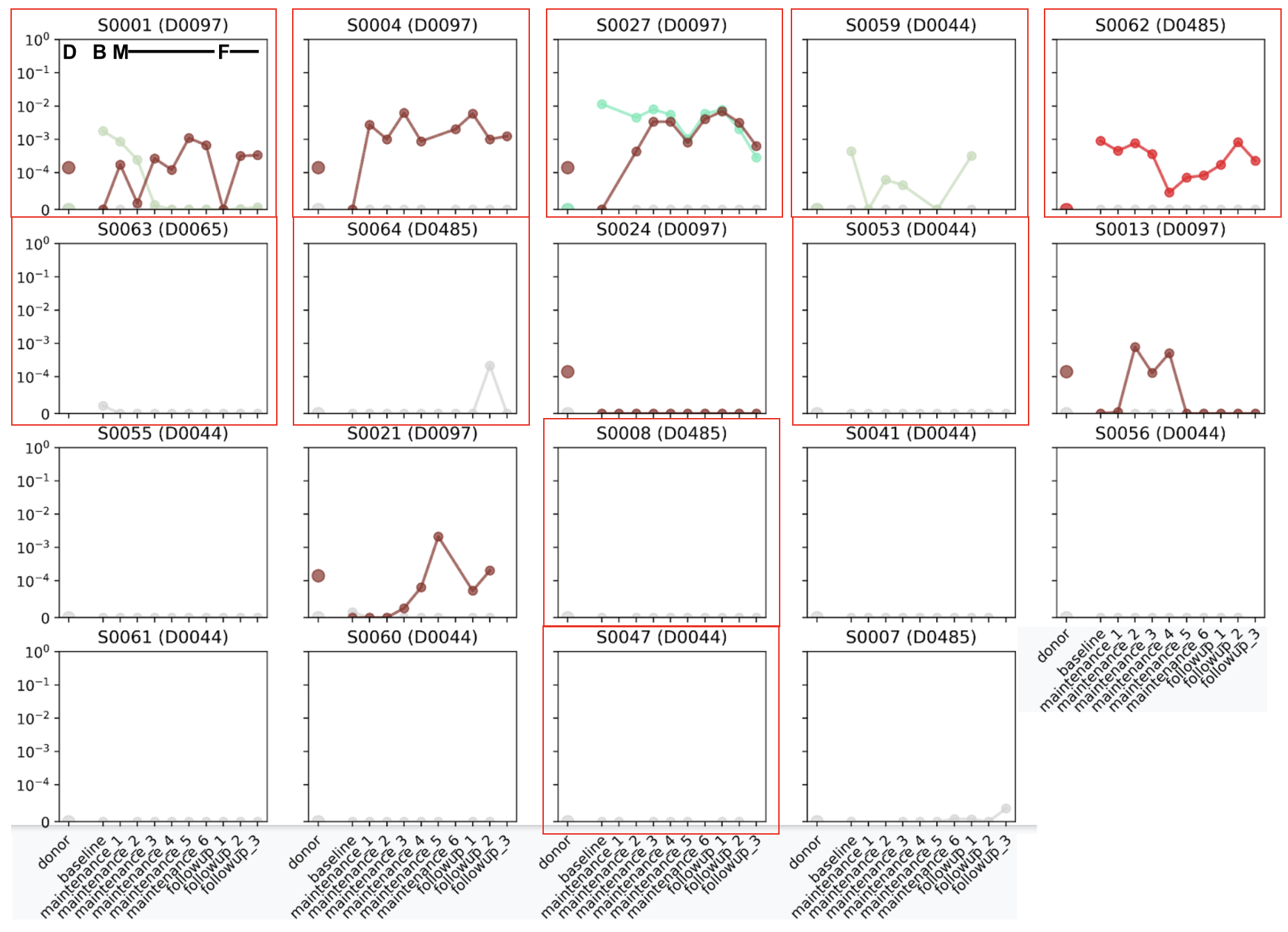


**Figure S10. Identification of strains for *Blautia hydrogenotrophica* in each patient across the clinical trial.** Colors indicate different strains as classified by StrainFacts; relative abundance of strains is displayed on the y-axis. Panels

corresponding to responders are indicated with a red box; *B. hydrogenotrophica* species-level counts (MetaPhlAn) were enriched at the first and/or third follow-up time point in subjects S0001, S0004, S0027, S0059, and S0062. The designated

donor for each subject is indicated in parentheses in panel titles. Disconnected points left of the time series for each subject indicate the mean relative abundance of strains across the relevant donor’s samples.

**
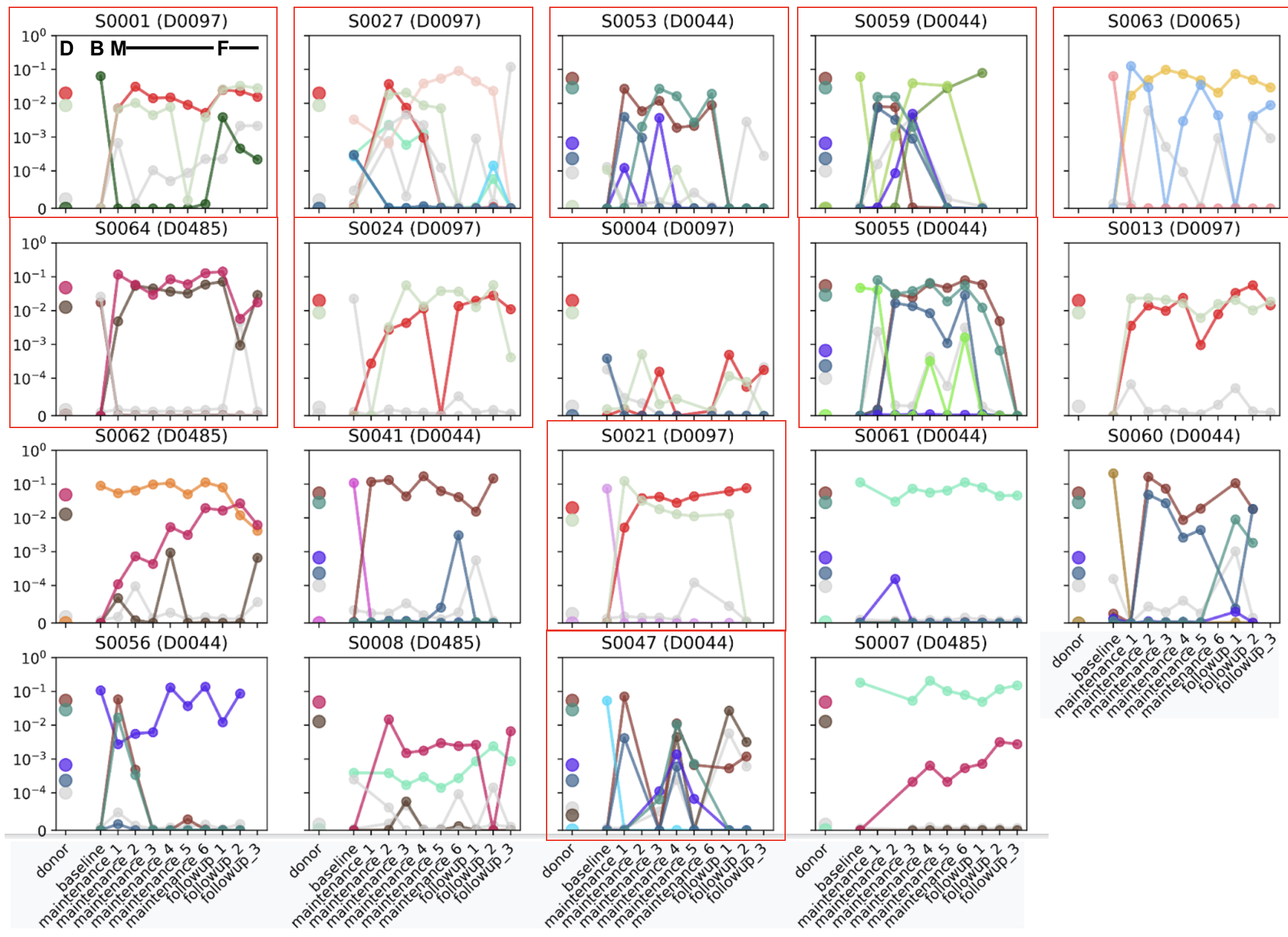
**

**Figure S11. Identification of strains for *Bacteroides dorei* in each patient across clinical trial time points.** Colors indicate different strains as classified by *StrainFacts*; relative abundance of strains is displayed on the y-axis. Panels corresponding to responders are indicated with a red box; *B. dorei* species-level counts (*MetaPhlAn)* were enriched at the first and/or third follow-up time point in subjects S0001, S0027, S0053, S0059, and S0063. The designated donor for each subject is indicated in parentheses in panel titles. Disconnected points left of the time series for each subject indicate the mean relative abundance of strains across the relevant donor’s samples.

**
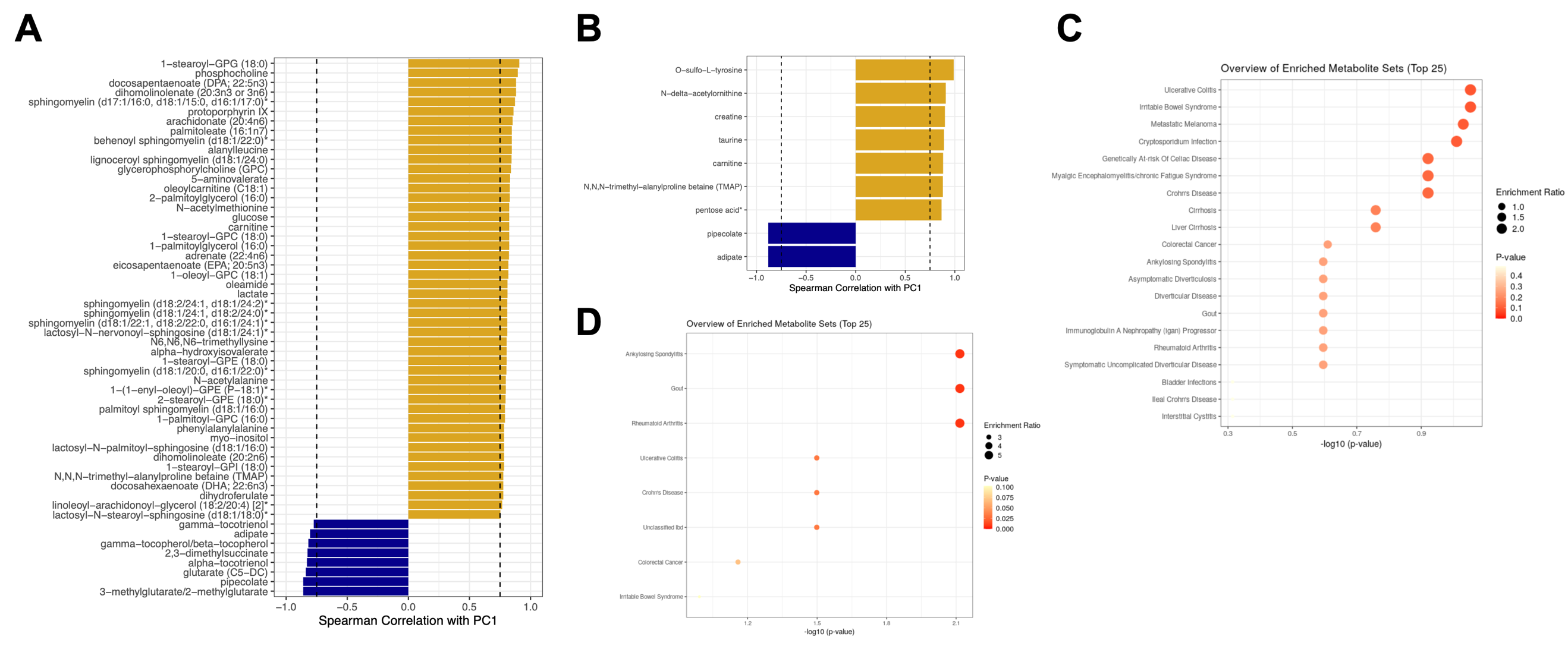
**

**Figure S12. Fecal metabolites associated with FMT responsiveness at the first and second follow-up time points. (A)** 56 and (**B**) 9 response-associated metabolites were identified (correlated with axis 1; **Fig. 4B-C**) at the first and second follow-up time points, respectively (DESeq2; FDR-adjusted p<0.05). Metabolites decreased and increased in responders are in yellow and blue, respectively. For the response-associated metabolites at the (**C**) first and (**D**) second follow-up, time points, metabolite enrichment analysis was performed using *MetaboAnalyst* to identify enriched metabolite sets known in disease.

**
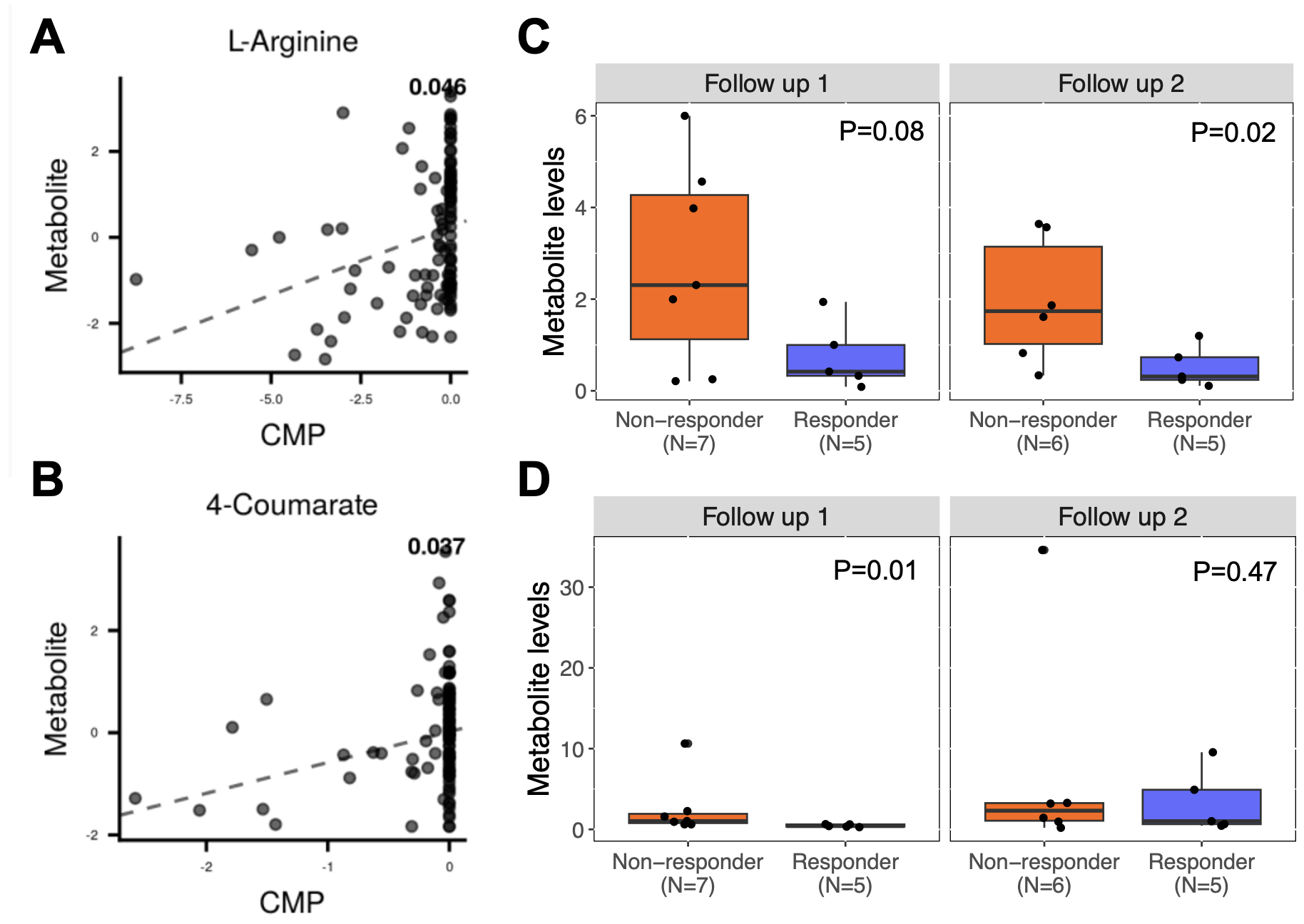
**

**Figure S13. Increased abundance of microbial metabolizers of fecal L-arginine and 4-coumarate associates with FMT responsiveness.** Integration of fecal shotgun

metagenomic (*HumaNn3*) and metabolomic datasets (Metabolon) was performed using *MIMOSA2* and identified **(A)** L-Arginine and (**B**) 4-coumarate as microbiome-derived

metabolites. Microbial metabolizers of both metabolites are shown in **Table S4**. (**C-D**) Normalized abundance levels for each metabolite are displayed in non-responders and responders at the first and second follow-up time points. Comparisons between non-

responders and responders were performed using a one-sided Wilcoxon rank-sum test. CMP; community metabolic potential score.

**
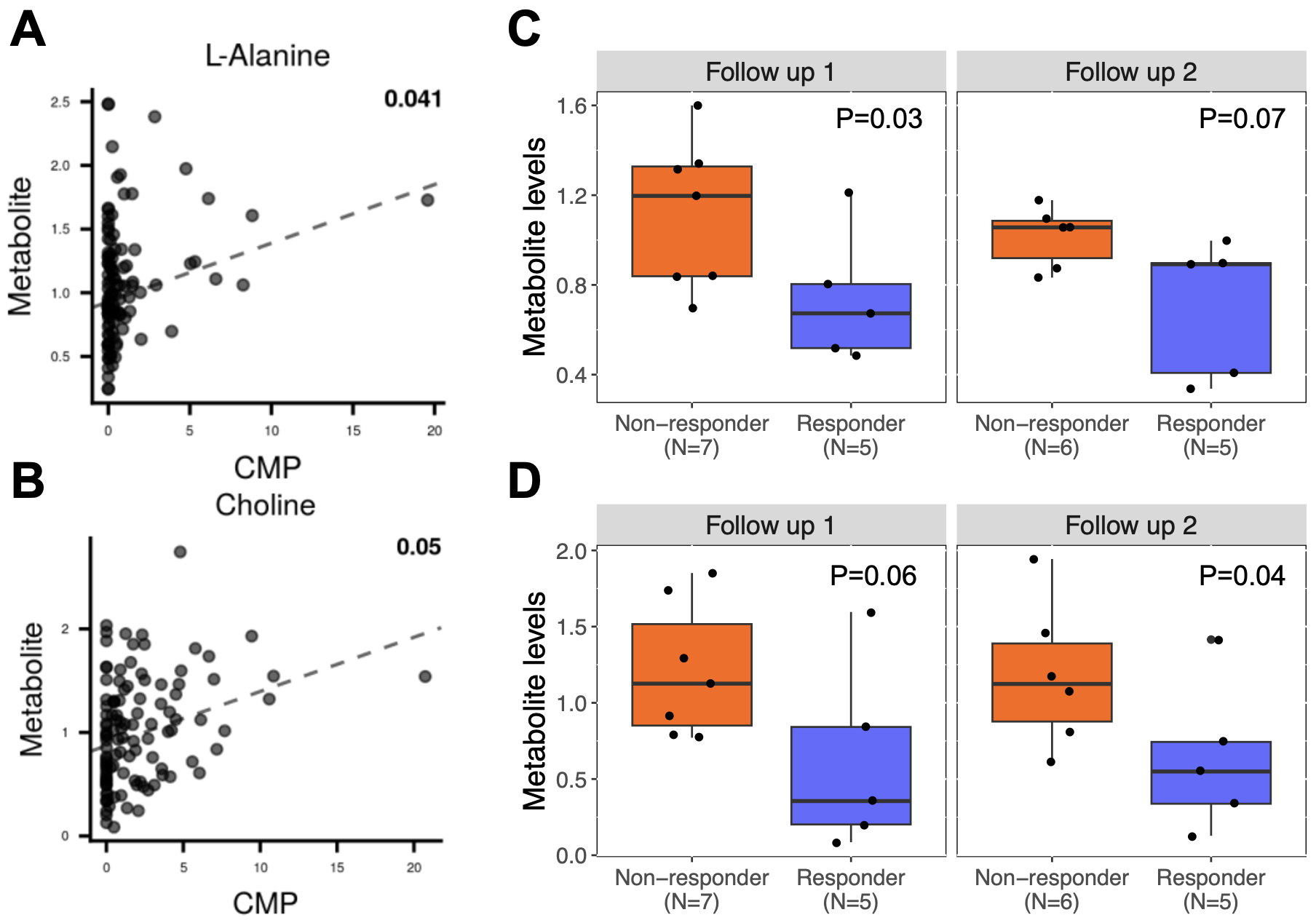
**

**Figure S14. Loss of microbial producers of fecal L-alanine and choline associates with FMT responsiveness.** Integration of fecal shotgun metagenomic (*HumaNn3*) and metabolomic datasets (Metabolon) was performed using *MIMOSA2* and identified (**A**) L-alanine and (**B**) choline as microbiome-derived metabolites. Microbial producers of both metabolites are shown in **Table S5**. (**C-D**) Normalized abundance levels for each metabolite are displayed in non-responders and responders at the first and second follow-up time points. Comparisons between non-responders and responders were performed using a one-sided Wilcoxon rank-sum test. CMP; community metabolic potential score.


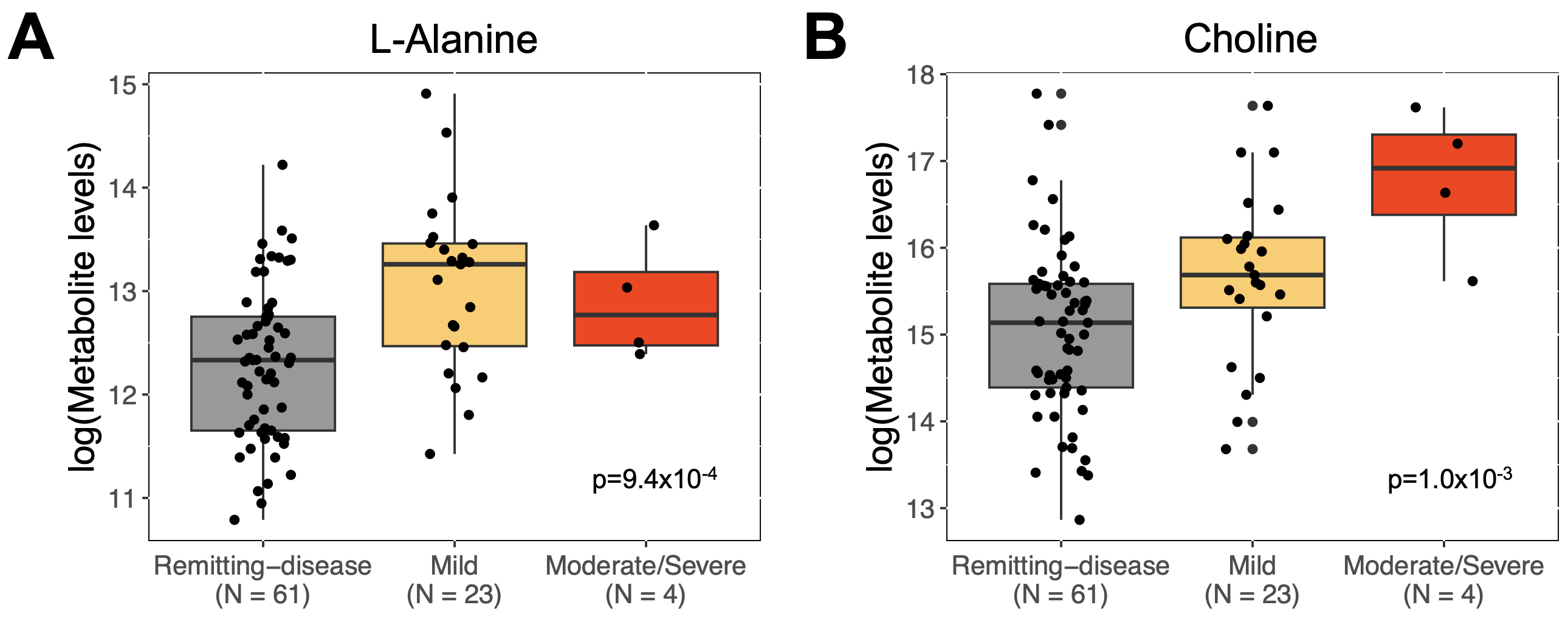


**Figure S15. Microbiome-mediated metabolites, L-alanine and choline, associate with severity of ulcerative colitis.** Metabolite abundance data were collected from The Inflammatory Bowel Disease Multi’omics Database Project. Log-transformed abundance levels for (**A**) L-Alanine and (**B**) choline were tested for an association with ulcerative colitis severity groups, as defined by SCCAI categories, using an ordinal logistic regression.
